# An Agrin–YAP/TAZ Rigidity Sensing Module Drives EGFR‐Addicted Lung Tumorigenesis

**DOI:** 10.1002/advs.202413443

**Published:** 2025-03-31

**Authors:** Reza Bayat Mokhtari, Divyaleka Sampath, Paige Eversole, Melissa Ong Yu Lin, Dmitriy A. Bosykh, Gandhi T.K. Boopathy, Aravind Sivakumar, Cheng‐Chun Wang, Ramesh Kumar, Joe Yeong Poh Sheng, Ellen Karasik, Barbara A. Foster, Han Yu, Xiang Ling, Wenjie Wu, Fengzhi Li, Zoë Weaver Ohler, Christine F. Brainson, David W. Goodrich, Wanjin Hong, Sayan Chakraborty

**Affiliations:** ^1^ Department of Pharmacology and Therapeutics Roswell Park Comprehensive Cancer Center 265 Elm and Carlton Streets Buffalo NY 14263 USA; ^2^ Institute of Molecular and Cell Biology 61 Biopolis Drive Proteos Singapore 138673 Singapore; ^3^ Department of Biostatistics Roswell Park Comprehensive Cancer Center Buffalo NY 14263 USA; ^4^ Center for Advanced Preclinical Research Frederick National Laboratory for Cancer Research National Cancer Institute NIH Bethesda MD 20892‐1088 USA; ^5^ Department of Toxicology and Cancer Biology Markey Cancer Center University of Kentucky Lexington KY 40536 USA; ^6^ Department of Medicine Roswell Park Comprehensive Cancer Center Buffalo NY 14263 USA; ^7^ Program of Developmental Therapeutics Roswell Park Comprehensive Cancer Center Buffalo NY 14263 USA

**Keywords:** agrin, EGFR, extracellular matrix, hippo pathway, lung cancer, YAP/TAZ

## Abstract

Despite epidermal growth factor receptor (EGFR) is a pivotal oncogene for several cancers, including lung adenocarcinoma (LUAD), how it senses extracellular matrix (ECM) rigidity remain elusive in the context of the increasing role of tissue rigidity on various hallmarks of cancer development. Here it is shown that EGFR dictates tumorigenic agrin expression in lung cancer cell lines, genetically engineered EGFR‐driven mouse models, and human specimens. Agrin expression confers substrate stiffness‐dependent oncogenic attributes to EGFR‐reliant cancer cells. Mechanistically, agrin mechanoactivates EGFR through epidermal growth factor (EGF)‐dependent and independent modes, thereby sensitizing its activity toward localized cancer cell‐ECM adherence and bulk rigidity by fostering interactions with integrin β1. Notably, a feed‐forward loop linking agrin–EGFR rigidity response to YAP–TEAD mechanosensing is essential for tumorigenesis. Together, the combined inhibition of EGFR–YAP/TEAD may offer a strategy to reduce lung tumorigenesis by disrupting agrin‐EGFR mechanotransduction, uncovering a therapeutic vulnerability for EGFR‐addicted lung cancers.

## Graphical abstract

The study identifies agrin‐EGFR mechanotransduction as a critical driver of lung adenocarcinoma which enhances EGFR signaling through an integrin‐FAK‐actomyosin dependent positive feedback on YAP/TAZ ‐TEAD in response to matrix stiffness. Targeting this oncogenic loop through combinatorial treatments inhibits lung cancer due to agrin impairment. Thus, agrin may serve as a biomarker for predicting response to these therapies.

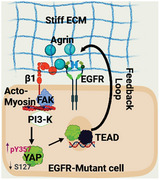

## 1 Introduction

The receptor tyrosine kinase (RTK) superfamily of cell‐surface receptors mediate cell signaling through extracellular growth factors within the tumor microenvironment (TME).^[^ ^1^ ^]^ As a member of the ErbB family of RTKs, the EGFR is frequently amplified and mutated in lung adenocarcinoma (LUAD), a prevalent non‐small cell lung cancer (NSCLC) subtype.^[^ ^2^ ^]^ EGFR amplifications or mutations including the in frame exon 19 deletion (Ex19Del) and exon 21 Leu858Arg point mutation (L858R) impact patient prognosis^[^ ^3^, ^4^ ^]^ In recent years, monumental progress has been achieved on EGFR targeting strategies, as exemplified by the small‐molecule inhibitors gefitinib and erlotinib, afatinib, and osimertinib, which revolutionized treatments for patients with advanced NSCLC harboring EGFR mutations, including the T790 M drug resistant mutation.^[^ ^5^ ^]^

Abnormal deposition of extracellular matrix (ECM) components creates a stiff environment that is increasingly being recognized for its regulatory yet essential role in a majority of the “cancer hallmarks.”^[^ ^6^ ^]^ ECM rigidity increases mechanosignaling associated with poor prognosis.^[^ ^7^ ^]^ Seemingly, these cell–ECM interactions can activate EGFR in cancer cells although the precise molecular mechanisms remain unclear,^[^ ^8^ ^]^ presenting a significant barrier toward developing effective targeted therapies for EGFR‐driven cancers. Consistent with the notion that ECM crosslinking, collagen deposition, and enhanced tissue rigidity activate integrin mechanosignaling,^[^ ^9^ ^]^ EGFR senses these changes in ECM rigidity.^[^ ^10^ ^]^ Despite intense investigation, a confounding caveat is the lack of knowledge surrounding how EGFR, or its mutants, interact with the corrupt ECM to promote lung cancer. Fueling this caveat is a binary problem whereby i) the identities of ECM protein(s) that mechanosensitize EGFR toward tumor stiffness remain elusive; and ii) the functional regulatory pathway of EGFR on these ECM proteins that crosstalk with tumor‐mechanotransduction network is not well defined.

The proteoglycan agrin, a ≈210 kDa proteoglycan, is naturally secreted in neuromuscular junctions (NMJ) and binds to its canonical receptors, lipoprotein related receptor‐4 (Lrp4), as well as a co‐receptor, muscle specific tyrosine kinase‐MuSK.^[^ ^11^ ^]^ Agrin is now shown to act as a key member of the corrupt ECM that mediates mechanosignaling in the liver TME, tumor‐mimicking wound‐healing microenvironment, and cardiac regeneration.^[^ ^12^ ^]^ Agrin, when overexpressed as a component of the corrupt liver carcinoma TME, hijacks Lrp4–MuSK in liver tumorigenesis.^[^ ^13^ ^]^ Agrin engages Yes‐associated protein (YAP), and its paralog TAZ (the converging effectors of the Hippo pathway) regulate cell growth, tumorigenesis, and mechanotransduction.^[^ ^13^, ^14^ ^]^ Serving as a mechanotransduction signal for YAP and TAZ, agrin nullifies the Hippo tumor‐suppressor pathway and thus promotes oncogenesis by sustaining tissue stiffness and maintaining integrin‐focal adhesion integrity.^[^ ^13a,e^ ^]^ In addition, secreted agrin from cancer cells stabilizes vascular endothelial growth factor receptor‐2 (VEGFR2) in an ECM stiffness‐dependent manner to support tumor angiogenesis and serve as biomarker for liver cancer.^[^ ^12^, ^15^ ^]^ Endorsing our initial discoveries, agrin has emerged as an oncogenic mediator in several solid cancers including pancreatic cancer, oral squamous cell carcinoma, and lung carcinomas.^[^ ^16^ ^]^ Further, agrin is activated by amyloid fibrils to initiate mechanosignaling through YAP/TAZ in melanomas.^[^ ^17^ ^]^ Despite these advances, the mechanistic insights into the oncogenic drivers that sustain agrin in the TME are lacking. Consistent with its overexpression and ability to stimulate PI3‐K and Notch signaling,^[^ ^16d,e^ ^]^ we identified agrin as one of the potential ECM gene co‐expressed with EGFR that can be indicative of a poor prognosis amongst LUAD patients. Intuitively, we probed the functional relevance of agrin–EGFR in approximating the stiff TME in lung tumorigenesis.

Here we reveal that EGFR governs tumorigenic agrin expression and then sensitizes the RTK to tumor‐stiffness through integrin mechanosignaling. The agrin–EGFR rigidity converges on YAP–TEAD (TEA‐domain transcription factors) mechanosensing, creating a feedforward loop that sustains agrin activity during tumorigenesis. Therefore, targeting the agrin‐EGFR‐YAP rigidity sensing module presents a promising therapeutic strategy for EGFR‐driven lung cancers.

## 2 Results

### 2.1 Co‐Expression of Agrin in EGFR‐Addicted LUAD

**Figure 1:**
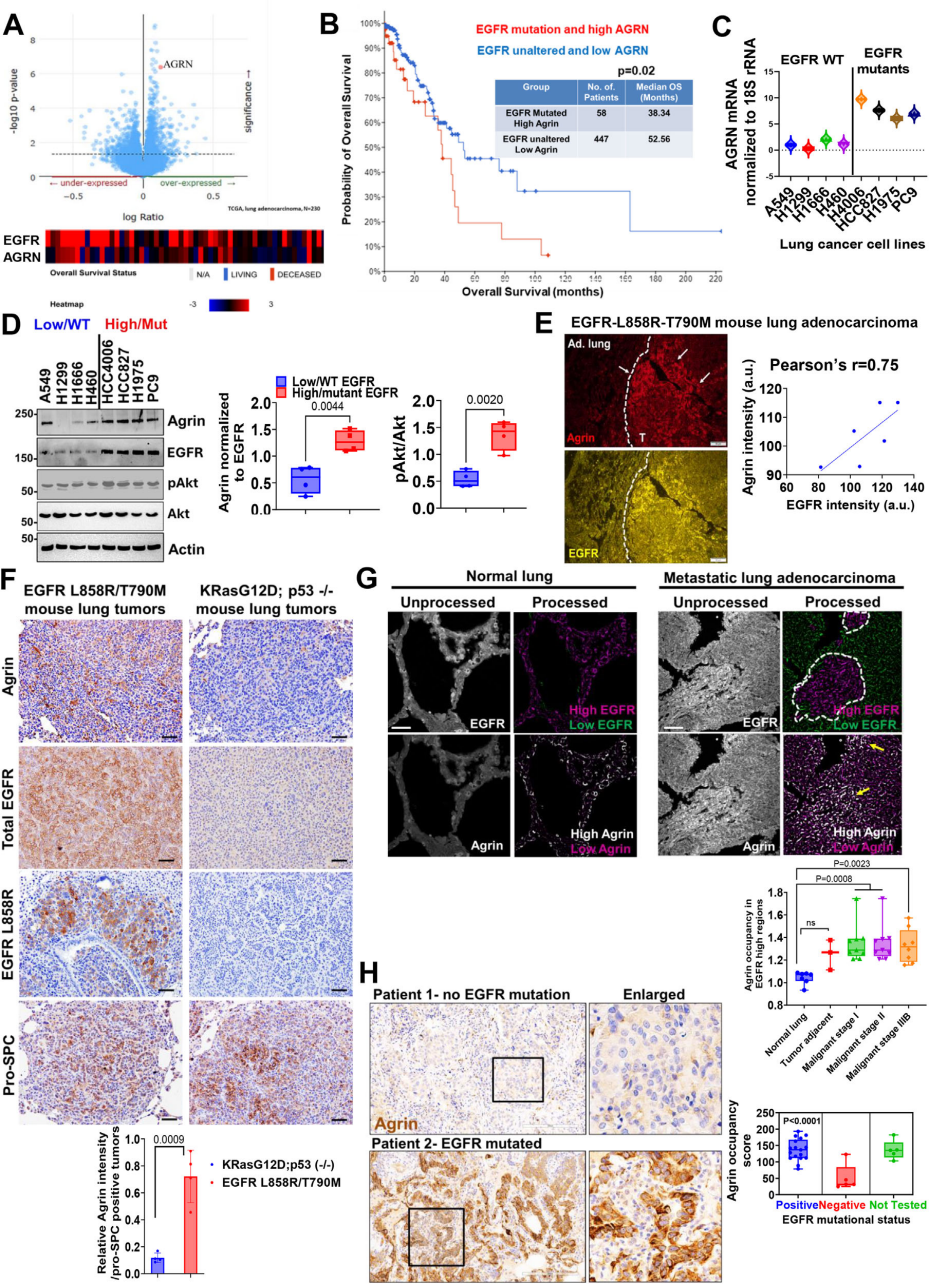
Agrin as a co‐dependent protein for EGFR‐driven lung cancers. A) Agrin (AGRN) as a top candidate gene overexpressed in lung adenocarcinoma with EGFR amplification and mutations (TCGA_dataset, *n* = 230). Inset shows the heatmap for the correlation of survival based on EGFR and AGRN expression. B) Survival curve based on high and low agrin–EGFR mutation in LUAD (TCGA_Firehose Legacy, *n* = 230, Log Rank Test, *p* = 0.02). C,D) RT‐PCR (C) and Western blot (D) showing agrin mRNA and protein levels in a panel of lung adenocarcinoma cell lines categorized based on low/wild‐type or high/mutant EGFR expression. Akt activity is shown in (D). β‐actin served as the loading control for panel D. The mean Agrin protein normalized to EGFR +/− SD and pAkt/Akt ratio are shown (*n* = 3, Students’ t test, *p* values indicated). E) Agrin and EGFR immunostaining in tumors extracted from EGFR–L858R–T790 M mouse lungs (*n* = 3 mice analyzed). Mean intensities for agrin and EGFR are plotted graphically (Pearson's *r* = 0.75). T‐tumor, ad.lung‐tumor adjacent lung tissue; white arrows point to regions with high agrin and EGFR. Scale bar: 50 µm. F) Representative immunohistochemistry (IHC) images for the indicated proteins in the lung tumors derived from EGFRL858R/T790 M and KRasG12D;p53 (–/–) models (*n* = 4 sections from four mice, Student's t test, *p* value indicated). G) Representative confocal microscopy images of normal and lung adenocarcinoma immunostained for EGFR and agrin. For EGFR (upper panels), regions of high expression are computationally determined as magenta, while those expressing low levels are in green. Likewise, high agrin expressing regions are in white while low expression is indicated by magenta (lower panels). Scale bar: 50 µm. Box and Whisker plots show the agrin occupancy normalized to EGFR levels (bars 1–99 percentile, central lines‐median, *n* = 7–10 tumors analyzed for each stage, one‐way ANOVA, Dunnett's multiple comparison test, *p* values indicated). H) Representative IHC analysis of Agrin from Roswell Park LUAD patient tissues with either no mutation or EGFR mutations (*n* = 27 patients). Boxed region presented as enlarged panels. The average Agrin occupancy score is presented as box‐and‐whisker plot (*n* = 27, Students t test, *p* value indicated, bars‐1‐99 percentile, central lines‐median).

To understand the molecular basis for EGFR's influence on the mechanoarchitecture of LUAD, we initially analyzed publicly available datasets to identify the potential ECM genes co‐expressed with amplified EGFR using Matrisome AnalyzeR. Upon stratifying these genes into several categories, we found that in addition to collagens, tenascin, and several other ECM‐associated proteins, agrin (AGRN) emerged as a top matrisome‐associated co‐expressed gene associated with high EGFR expression (**Figure** **1**A; Figure S1A, Supporting Information). Notably, agrin was overexpressed in LUAD when compared with adjacent normal tissues and its expression was correlated with poor overall survival (OS) and disease recurrence (Figure S1B,C, Supporting Information). Furthermore, in LUAD patients with EGFR mutations, higher expression of agrin was linked to poor OS (Figure 1B), aligning with the recent findings that identified a role of agrin in NSCLC.^[^ ^16d,e^ ^]^ Importantly, lung cancer cell lines with EGFR mutations or exhibiting constitutive activation of downstream signaling exemplified by higher Akt activation showed elevated agrin mRNA and protein levels that correlated with EGFR status (Figure 1C,D). Interestingly, agrin mRNA is positively correlated with expressions of EGFR and ERRB2 family of RTKs but was insignificant when compared to other RTKs (e.g., PDGFRA, KDR, IGFR) or KRAS (Figure S1D,E, Supporting Information). This correlation was subsequently validated in a mouse model driven by hEGFR–L858R which spontaneously develops LUAD,^[^ ^18^ ^]^ and where agrin expression was restricted to EGFR‐driven tumors (Figure 1E). Because patients with EGFRL858R mutations treated with EGFR inhibitors often manifest T790 M secondary mutations, we analyzed if agrin expression was persistent in the mouse lungs by the autochthonous hEGFR‐T790M/L858R^LSL^ activation.^[^ ^19^ ^]^ Higher agrin expression was correlated with EGFR and pro‐surfactant protein C (pro‐SPC), a marker for tumors derived from AT2 cell types (Figure 1F). Interestingly, the distribution of agrin was significantly lower in adenocarcinomas generated in KRAS‐G12D, p53 deficient mice lungs that exhibited reduced EGFR with no evidence of L858R mutations (Figure 1F). This observation aligned with the clinical data that KRAS mutant LUAD patients with did not present any differences in their OS regardless of agrin levels (Figure S1F, Supporting Information). In line with the in vitro and in vivo evidence, we confirmed the co‐expression of agrin–EGFR in LUAD patients of diverse demographics. First, we noted that agrin expression was predominantly localized to regions of higher EGFR expression within LUAD tissues (Figure S2A, Supporting Information). Subsequently, we developed a computational image analysis pipeline to identify regions of high and low EGFR and correlated these with agrin expression status within normal and cancerous lung tissues (Figure 1G). A diffuse and low expression of agrin coupled to uniform EGFR expression was observed in normal lung tissues (Figure 1G). Notably, distinct regions of high EGFR expression were correlated with elevated agrin expression (white clusters) were identified across different malignant grades of LUAD (Figure 1G). Second, the agrin–EGFR co‐expression observed in various pathologic grades of LUAD pertained to solid, papillary, and acinar adenocarcinomas but was not obvious in small cell lung cancers from an Asian cohort (Figure S2B, Supporting Information). LUAD patients from the Roswell Park repository who did not harbor EGFR mutations exhibited low Agrin expression, whereas significantly increased levels of agrin were observed in those harboring EGFR mutations (Figure 1H). These findings suggest that EGFR‐driven lung malignancies are specifically enriched with high agrin expression.

### 2.2 EGFR Induces the Tumorigenic Agrin Expression

**Figure 2:**
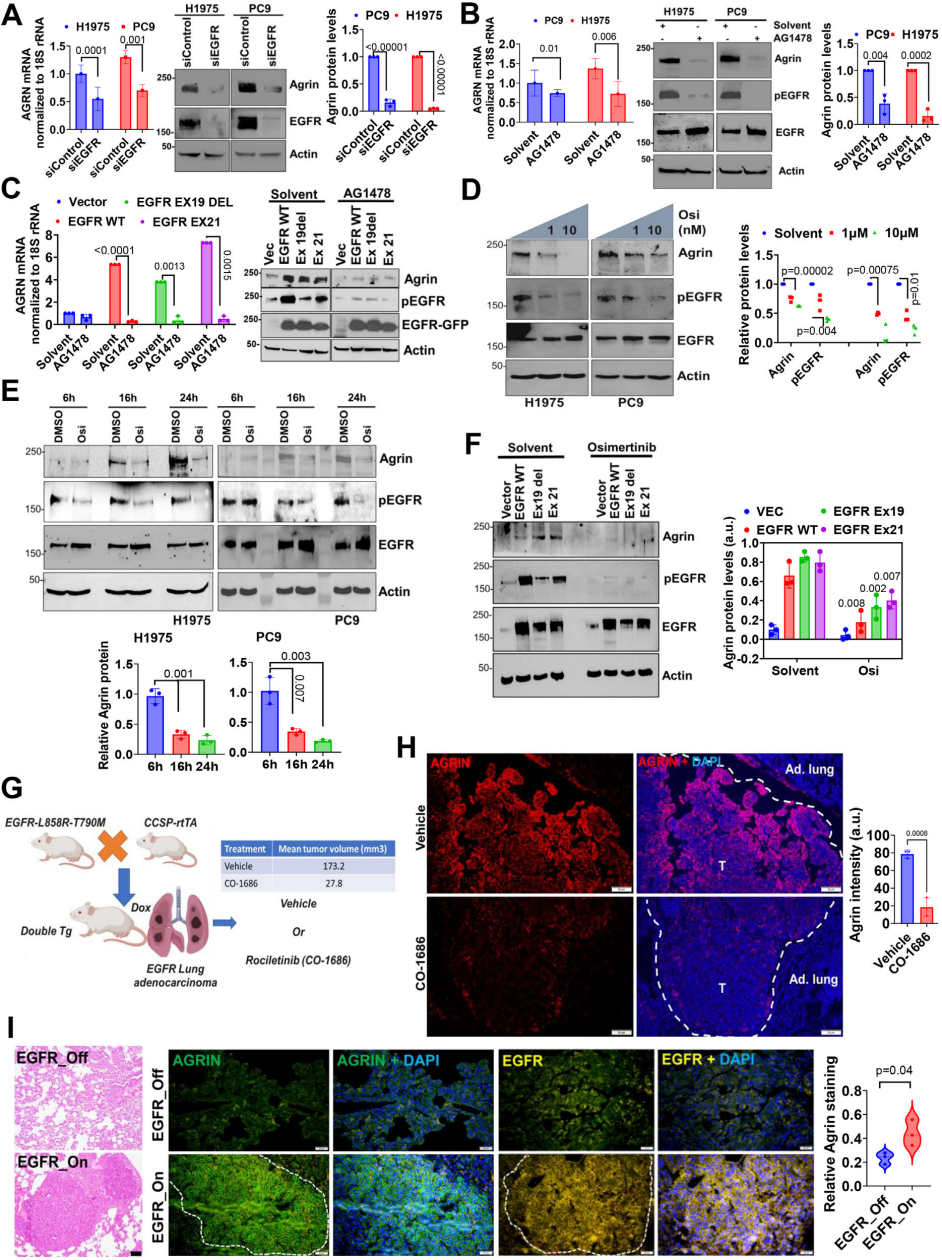
EGFR controls tumorigenic agrin expression. A) RT‐PCR analysis (left) and Western blot (right) for agrin mRNA and protein levels in indicated cell lines treated with EGFR siRNA. β‐actin served as the loading control. Agrin protein densities± SD is quantified (*n* = 3, Students t test, *p* values indicated). B) RT‐PCR analysis (left) and Western blot (right) for agrin mRNA and protein levels in indicated cell lines treated with 1 µM AG1478 for 18 h. In the right panel, pEGFR levels indicate targeted action of AG1478 while total EGFR and β‐actin served as controls. Agrin protein densities ±SD were quantified (*n* = 3, Students t test, *p* values indicated). C) RT‐PCR analysis of agrin mRNA in H1299 expressing vector, EGFR WT, or its mutants EX19 DEL and EX21 treated with 1 µM AG1478 for 18 h (left panel). Cells treated similarly were analyzed for agrin, pEGFR, and GFP by Western blot. β‐actin served as the loading control (*n* = 3 biological repeats). (E) (D) The indicated cell lines were treated with increasing doses of osimertinib for 18 h. Cell lysates were analyzed by Western blot for agrin, pEGFR, and EGFR, respectively. β‐actin served as the loading control. Relative agrin and pEGFR proteins were quantified (*n* = 3, multiple t test, *p* values indicated). E,F) H1975, PC9, and H1299 expressing EGFR, or its mutants were treated with 10 nM osimertinib for the indicated time. Resulting cell lysates were analyzed for the same proteins as in panel (F). Data presented as mean agrin protein intensity ± SD normalized to actin (H) (*n* = 3 repeats, Students t test, *p* values indicated) (G) Schematic showing the EGFR–L858R–T790 M based mouse lung adenocarcinoma model. Mean tumor volume (imaged by MRI) of mouse lungs treated with vehicle or CO‐1686 is presented (*n* = 3 animals/group). H) Representative confocal microscopy images showing agrin expression in mouse lung tumors from (I). T‐tumor; Ad.lung‐adjacent lung (*n* = 3 animals/group, data presented as the mean +/− SD, Student's t test, *p* value is indicated). Scale bar: 50 µm. I) Adenovirus‐Cre‐mediated activation of EGFR–L858R–T790 M showing the development of tumors in mouse lungs, as represented by Hematoxylin–Eosin‐stained images (left). Representative confocal microscopy images showing agrin expression in mouse lungs. Dashed region‐Tumor. The mean +/− SD agrin intensity is shown (*n* = 3 animals per group, Student's t test, *p* value indicated). Scale bar: 20 µm.

The co‐expression of agrin with EGFR and its mutations prompted us to assess if EGFR is a regulator of agrin expression during tumorigenesis. Utilizing the Cancer Dependency Map (DepMap), we first analyzed whether the loss of EGFR (via CRISPR Public 22Q4 datasets) in over 1000 cancer cell lines requires agrin as a co‐dependent gene for regulation of cellular proliferation (Figure S3A, Supporting Information). Indeed, EGFR knockout cell lines exhibited a higher dependency on agrin as indicated by the negative enrichment scores suggestive of loss in proliferation (Figure S3A, Supporting Information). Likewise, silencing EGFR in H1975 (harboring the sensitive L858R and resistant T790 M mutations) and PC9 (with a deletion in exon 19 of the EGFR) cell lines significantly reduced the mRNA and protein levels of agrin (**Figure** **2**A). Since these LUAD cell lines are sensitive to EGFR TKIs, we exposed them to various EGFR inhibitor treatments to assess their impact on agrin expression. Consistently, AG1478 (Tyrphostin) significantly inhibited the mRNA and protein expression of agrin (Figure 2B). AG1478 treatment strongly inhibited the EGFRpY1068 phosphorylation without affecting its total levels, suggesting that impaired EGFR signaling by AG1478 resulted in reduced agrin expression (Figure 2B). Next, we overexpressed wild‐type EGFR and exon 19‐del and exon 21‐point mutant versions in H1299‐GFP cells that have low endogenous EGFR levels.^[^ ^20^ ^]^ Both wild‐type or mutant EGFR increased the mRNA and protein levels of agrin, which were inhibited by short‐term AG1478 treatments (Figure 2C). In an interesting observation, while employing DepMap, we noted that several cancer cells that were sensitive to osimertinib had a negative score for agrin in addition to phosphorylated EGFR (Figure S3B, Supporting Information). Osimertinib treatment significantly reduced agrin expression in a dose‐dependent manner (Figure 2D), with maximal reduction within 16–24 h post‐treatment (Figure 2E). The apparent increase in agrin protein levels in the DMSO‐treated controls at 24 h was likely due to the time‐dependent accumulation of Agrin in response to an extremely stiff matrix as experienced on plastic plates. Moreover, the enhanced expression of agrin induced by either wild‐type or mutant EGFR was severely compromised by Osimertinib treatment (Figure 2F). In a spontaneous lung adenocarcinoma generated by the expression of the EGFR L858R in type II pneumocytes under the control of doxycycline (Dox),^[^ ^21^ ^]^ induction of EGFR activated agrin expression within the multifocal adenocarcinomas when compared with adjacent or non‐induced lung tissues (Figure 2G,H). Rociletinib (CO‐1686), an orally active EGFR inhibitor, significantly reduced the lung tumor incidence rates in these mice.^[^ ^22^ ^]^ In line with our in vitro findings, loss of agrin expression in the remaining tumor nodules after CO‐1686 treatment was observed in vivo (Figure 2G,H). Additionally, adenovirus expressing Cre‐recombinase that led to the expression of EGFR‐L858R/T790 M in mouse lungs formed spatially diffused lung adenocarcinoma with increased agrin expression (Figure 2I). Together, these findings reveal that EGFR, and its oncogenic mutations dictate tumorigenic agrin expression.

### 2.3 Agrin Confers Matrix Rigidity‐Dependent Oncogenic Attributes to EGFR

**Figure 3:**
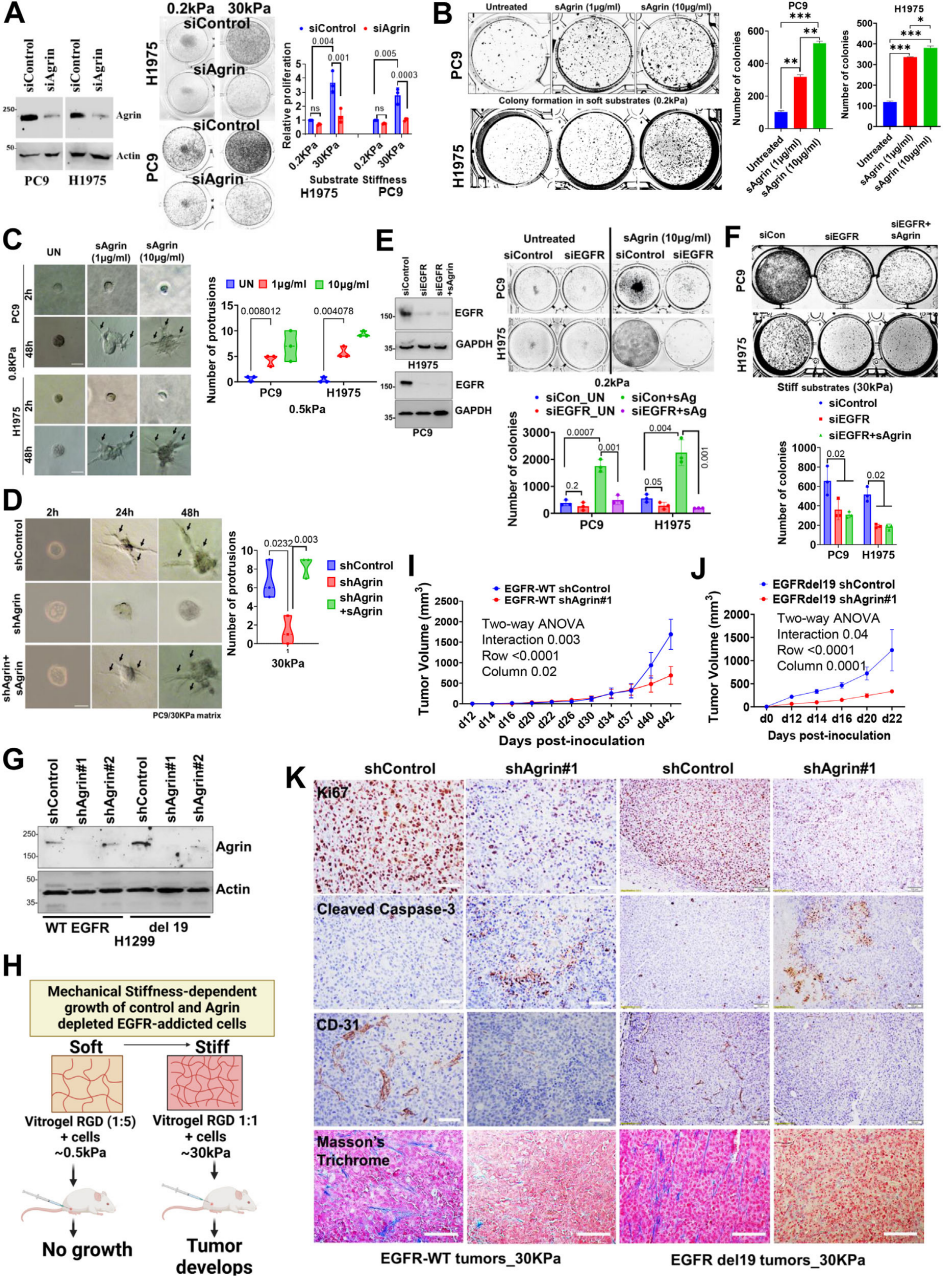
Agrin bestows ECM‐stiffness‐oncogenic traits to EGFR‐addicted cancer cells. A) Western blot validating agrin knockdown in the indicated cell lines (left). 10 000 cells were plated on 0.2 or 30 kPa substrate and analyzed for colony formation after day 5. Representative colony images are shown, and mean number of colonies±SD were quantified using ImageJ (*n* = 3, multiple t test, *p values* indicated). B) Indicated cell lines cultured on 0.5 kPa substrates were treated with increasing doses of sAgrin for 5 days. Representative colony images are shown. Results were quantified as in panel (A) (*n* = 3, data presented as the mean +/− SD, Student's t test, *p = 0.03, ***p = 0.004, 0.001, **p = 0.02, respectively). C,D) Indicated cell lines were 3D‐cultured in VitroGel matrix corresponding to 0.5 kPa either alone or containing increasing concentrations of sAgrin for 48 h (C). The shControl, agrin‐depleted cells, and those treated with 10 µg/mL sAgrin were cultured on 30 kPa stiff collagen matrix for 2 days. Representative bright‐field images of tumor spheres are shown for both panels. The number of protrusive structures is quantified for each condition (experiment repeated three times, *n* = 3 spheres analyzed for each group, Student's t test, *p* values indicated). E,F) Western blot validating EGFR knockdown in the indicated cell lines. Representative colony images of control, EGFR silenced, and those treated with 10 µg/mL sAgrin on 0.2 kPa (E) and 30 kPa (F) (*n* = 3, mean colony number±SD, Students t test, *p* values shown). G) Western blot validating the loss of agrin in H1299 EGFR WT and Ex19DEl. β‐actin served as the loading control. H) Schematic showing the stiffness‐based tumorigenic model for control and agrin depleted EGFR‐addicted cells. I–J) Tumor volumes of shControl and shAgrin EGFR WT and EX19DEL subcutaneously injected with stiff VitroGel in NOD/SCID mice (*n* = 5 animals/group; data presented as the mean +/− SEM, two‐way ANOVA, *p* values indicated). (K) Representative images of Ki67, cleaved caspase‐3, and CD‐31 immunohistochemistry in EGFR WT and Del19 control and agrin‐depleted tumors (*n* = 3 tumors per condition). Scale bar: 100 µm.

Though agrin orchestrates the mechanobiological environment of tumorigenesis and wound healing,^[^ ^12a^ ^]^ its functional impact on EGFR remains elusive. We, therefore, first tested whether agrin bestows stiffness‐dependent oncogenic potential in EGFR‐responsive lung cancer cell lines. Direct comparisons of cell proliferation revealed that substrate stiffness favored the growth of PC9 and H1975 cells (**Figure** **3**A). Agrin depletion in these cell lines significantly reduced their clonogenic potential on stiff matrix which was less obvious on compliant conditions (Figure 3A). In a complementary set of experiments, sAgrin (the recombinant C‐terminal fragment of agrin representing soluble agrin) enhanced clonogenic potential of these cells in compliant matrices (Figure 3B). Treatment with an agrin function‐blocking antibody reduced the proliferative rates in stiff matrix (Figure S3C, Supporting Information), while agrin knockdown significantly reduced the migratory and invasive potential of these cells (Figure S3D,E, Supporting Information). Next, we generated agrin knock‐out PC9 cells (Figure S3F, Supporting Information), out of which KO#64 cell clone validated the impaired EGFR downstream signaling that accounted for reduced proliferative rates (Figure S3F,G, Supporting Information). Further, sAgrin supported protrusive structures in addition to increased spheroid size in soft substrates in 3D cultures (Figure 3C). Likewise, the enhanced growth and protrusive structures of PC9 and H1975 spheroids in stiff matrix were abolished by agrin depletion but significantly rescued when sAgrin was nourished in the stiff matrix (Figure 3D). Interestingly, knock down of EGFR significantly suppressed the proliferative potential of EGFR‐mutant cell lines induced by sAgrin on compliant and stiff matrices, which could not be rescued by sAgrin, thus indicating that Agrin‐tuned matrix rigidity converges on EGFR to regulate cancer cell proliferation (Figure 3E,F). Furthermore, in contrast to EGFR‐reliant cells, silencing agrin in H1666 and HL460 cell lines that have low endogenous WT EGFR did not affect their proliferation rates under stiff conditions (Figure S3I, Supporting Information). In light of the notion that EGFR addiction results in a pro‐oncogenic outcome, we reasoned that agrin‐induced stiffness cues acting on EGFR, in part, underlies the EGFR‐driven oncogenicity attributed due to increased EGFR. To assess this in a scenario that mimics EGFR‐addicted tumor‐thriving conditions, we silenced agrin in both wild‐type and mutant EGFR (del 19) overexpressing H1299 (Figure 3G) and implanted them subcutaneously with soft (≈0.5 kPa) or stiff VitroGel RGD (30 kPa) in SCID mice (Figure 3H). Interestingly, no tumor growth was achieved when cells were mixed with soft VitroGel, suggesting that increased matrix rigidity is required for tumor growth. In contrast, the tumor growth from shControl wild‐type and mutant EGFR overexpressing cells supported by stiff VitroGel were significantly reduced upon agrin silencing (Figure 3I,J). This was accompanied by a reduced proliferative index (Ki67) and collagen meshes but increased cleaved caspase‐3 in agrin‐depleted tumors (Figure 3K). Cumulatively, these findings indicate that agrin‐driven ECM may act as an upstream player that relays matrix rigidity signals to ensure EGFR signaling for tumorigenesis.

### 2.4 Agrin Mechanoactivates EGFR

**Figure 4:**
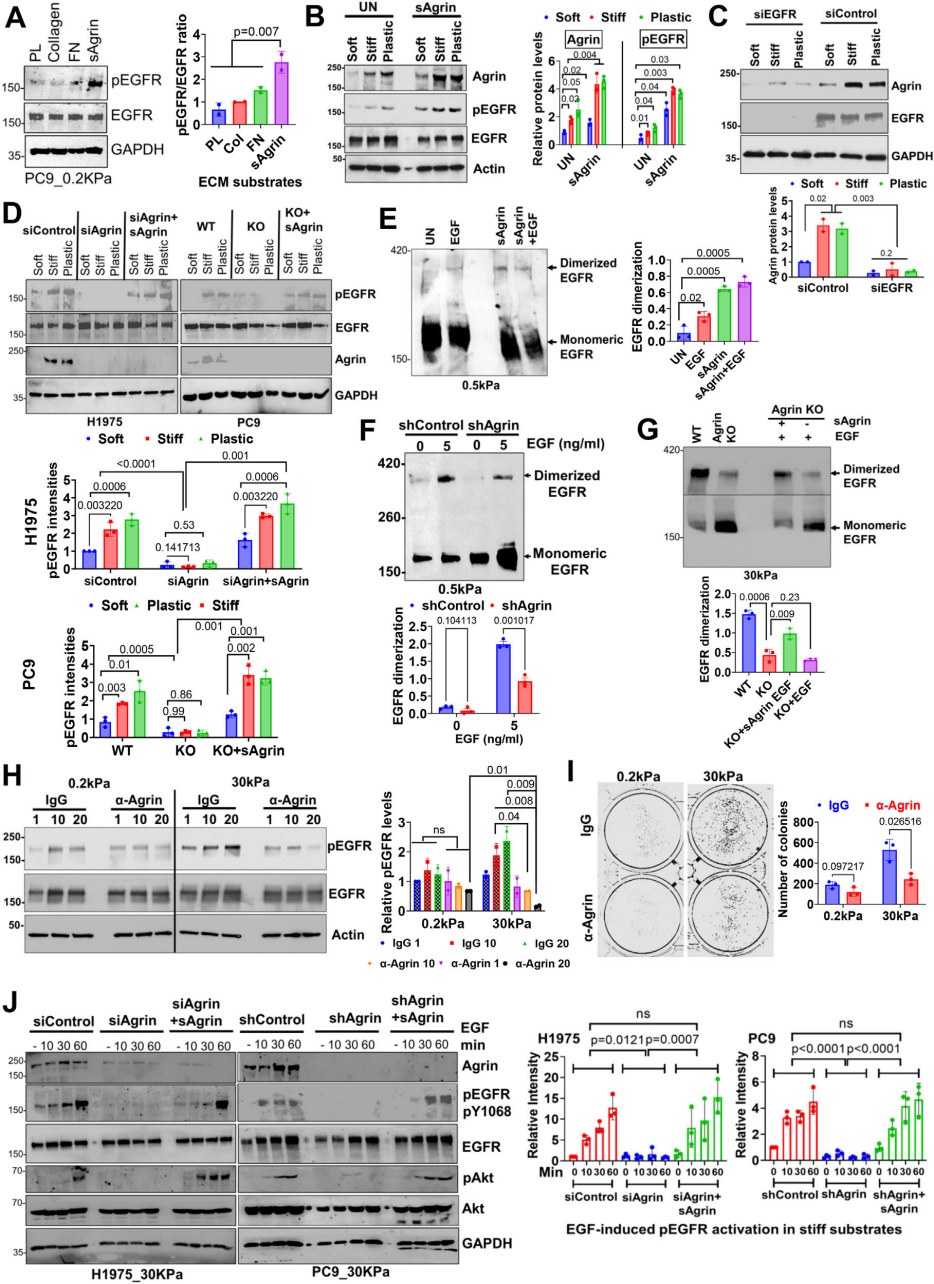
Agrin conveys ECM‐rigidity responses to EGFR. A) Western blot analysis showing EGFR activity of PC9 cells seeded on 0.2 kPa with 10 µg/mL indicated ECM agents for 1 h. Mean agrin intensity±SD was quantified (*n* = 3, One‐Way ANOVA, *p* values indicated). B) Untreated PC9 cells were seeded on soft (0.5 kPa), stiff (30 kPa), or plastic dishes either alone or containing 10 µg/mL sAgrin for 1 day. Cell lysates were analyzed by Western blot for the indicated proteins. GAPDH served as the loading control. Mean Agrin and pEGFR intensities±SD presented as heat map (*n* = 3, Multiple t tests, *p* values indicated). (C) Control and EGFR‐silenced H1975 on indicated substrates are assessed for Agrin and EGFR expression by Western blot. Mean agrin protein±SD levels were quantified (*n* = 2, multiple t tests, *p* values indicated) (D) Control and agrin siRNA were seeded similarly as in (B) either alone or in the presence of sAgrin in the substrates. Cell lysates were analyzed as in (B). Mean pEGFR intensities±SD presented (*n* = 3, multiple t tests, *p* values indicated). (E) Western blot analysis of PC9 lysates on 0.5 kPa treated with EGF (5 ng/mL), sAgrin (1 µg/mL), or their combination for 30 min and then treated with bis(sulfosuccinimidyl) suberate (BS3; 2 mM) for 30 min. The ratio of dimerized/monomer densities are presented as mean±SD (*n* = 3, multiple t tests, *p* values indicated). (F) Control and agrin knockdown PC9 seeded on 0.5 kPa and treated with 5 ng/mL EGF for 30 min and processed and quantified as in panel (E) (*n* = 3, Multiple t tests, *p* values shown). (G) Wild‐type (WT) or agrin KO PC9 seeded on 30 kPa treated with EGF or sAgrin as in (E‐F). Whole cell lysates were probed by Western blot for the EGFR as in (D). Results quantified as in panel (E). H) Western blot analysis of whole cell lysates of H1975 on 0.2 and 30 kPa containing indicated concentrations of sAgrin either treated with an isotype control (black dotted) or anti‐agrin antibody for 4 h. β‐Actin served as the loading control. Mean pEGFR±SD levels are presented (*n* = 3, Students’ t tests, *p* values indicated). (I) Colony formation of H1975 treated with isotype or agrin antibodies for 5 days on 0.2 or 30 kPa substrates. The mean number of colonies±SD are shown (*n* = 3, Multiple t tests, *p* values indicated). J) Western blot analysis of cell lysates treated with EGF for indicated time post‐serum starvation and analyzed for the indicated proteins (*n* = 3 repeats, data quantified as the mean pEGFR band intensity +/− SD, Student's t test, *p* values indicated).

Considering the impact of tissue rigidity on tumorigenesis,^[^ ^6^, ^9^ ^]^ we reasoned that agrin functions as a mechanotransducer, relaying rigidity responses to sustain EGFR activation and signaling. This hypothesis is supported by recent observations that matrix rigidity regulates the activity of EGFR and its downstream signaling.^[^ ^10^, ^23^ ^]^ Because soft substrates are not conducive to the activation of EGFR,^[^ ^10^ ^]^ ECM supplemented with sAgrin rapidly promoted EGFR phosphorylation even on soft substrates (**Figure** **4**A). Interestingly, none of the other tested ECM components including collagen or fibronectin (FN) activated EGFR in soft matrix, suggesting that agrin preferentially transmits ECM rigidity response to EGFR (Figure 4A). Indeed, agrin mRNA levels increased with substrate rigidity and this was further amplified in the presence of sAgrin in the matrix (Figure S4A, Supporting Information), indicating that bulk mechanical rigidity induces agrin expression. Consistent with the increased Agrin protein levels, the incremental change in activated EGFR in response to varying rigidity (from soft to very stiff plastic plates) was substantially increased in the presence of sAgrin in the matrix (Figure 4B). Consistently, silencing EGFR strongly suppressed the increased agrin protein levels observed with matrix rigidity (Figure 4C). In the absence of EGF, sAgrin treatment in compliant matrices and 3D‐Matrigel activated EGFR in a dose‐dependent manner within 30 min (Figure S4B,C, Supporting Information). This response was further accentuated by the presence of EGF (Figure S4C, Supporting Information). Considering that EGFR activation may occur in the absence of EGF, we evaluated the impact of agrin and ECM‐stiffness on EGF secretion as an alternate mechanism associated with EGFR activation. However, agrin silencing or supplementation with sAgrin in EGFR‐mutant and WT cells did not alter EGF levels in compliant or stiff conditions (Figure S4D, Supporting Information), therefore, allowing us to rule out the possibility of initiating EGFR signaling via autocrine activation of EGF by agrin upon increased tissue rigidity. The basal EGFR activity is increased with substrate stiffness, which was drastically reduced in agrin‐silenced or KO cells (Figure 4D). Supplementing sAgrin to soft, stiff, or very stiff (plastic) substrates readily rescued EGFR phosphorylation (Figure 4D). Since homodimerization activates phosphorylation at residue Tyr1068 of EGFR to promote a downstream signaling,^[^ ^24^ ^]^ and considering that ligand‐independent homodimerization of EGFR is also plausible with unknown impacts on mechanotransduction,^[^ ^25^ ^]^ we next probed if agrin was necessary and sufficient to trigger EGFR homodimerization and phosphorylation in response to matrix stiffness using previously established protocol.^[^ ^26^ ^]^ In the soft matrix, EGFR homodimerization remained low, though EGF exposure for 30 min expectedly increased EGFR homodimerization (Figure 4E). Strikingly, sAgrin‐enriched compliant matrices rapidly led to the induction of EGFR dimerization either independently or synergistically with EGF, which exceeded over 3‐fold that with EGF alone (Figure 4E). Despite minor reductions in monomeric forms, the impact of sAgrin combined with EGF on EGFR dimerization was similar to that induced by sAgrin, raising the possibility of multiple forms of EGFR in combination with EGF due to matrix crosslinking which likely is beyond the resolution of BS3 crosslinking assay. Because soft substrates are not naturally conducive to EGFR dimerization, EGF treatment induced dimerization of EGFR in compliant matrices without impacting monomeric forms in control cells. In such scenarios, a mild reduction of homodimerized EGFR upon EGF addition was observed in agrin knockdown cells (Figure 4F). In contrast, EGFR dimerization was more severely compromised when agrin was silenced in cells cultured on stiff matrix and was readily rescued by sAgrin treatment, an effect which was not rescued by EGF treatment alone (Figure 4G). While EGF may induce EGFR activation irrespective of matrix stiffness, the increased agrin expression with tissue stiffness markedly influenced EGFR activity in the presence of EGF. Consistently, EGF‐induced EGFR phosphorylation and downstream Akt activation were inhibited by agrin silencing in EGFR‐mutant cells on soft substrates which was not obvious in EGFR‐WT cells that had lower overall EGFR (Figure S4E, Supporting Information). Moreover, agrin knockdown preferentially impacted EGF‐induced Akt activation when compared with p42/44 MAPK signaling in soft substrates (Figure S4E, Supporting Information). As expected, compared to H1666 cells having wildtype EGFR, sAgrin substantially stimulated pEGFR‐Akt signaling compared to p42/44 MAPK pathway in PC9 cells having mutant EGFR, which was further heightened in the presence of EGF (Figure S4E, Supporting Information). These results suggest that the ability of agrin to mechanoactivate EGFR and downstream PI3‐K‐Akt signaling in compliant matrix is preferential to EGFR mutant cell lines (Figure S4E, Supporting Information), although the detailed mechanism is yet to be defined. While agrin‐silencing did not inhibit EGFR activity in WT‐cells, EGF‐treatment slightly increased agrin expression in EGFR‐WT cells when compared to EGFR‐Akt signaling (Figure S4F, Supporting Information), implying that enhanced EGF or other oncogenic ligands may have a minor impact on agrin expression in non‐EGFR‐mutant conditions. Treatment with an agrin function‐blocking antibody caused a striking loss of EGFR activity in a dose‐dependent manner in stiff matrix but remained less effective on soft matrices (Figure 4H). Consistently, the ECM‐stiffness‐induced proliferation was significantly inhibited by anti‐agrin antibody on stiff substrates when compared to compliant substrates (Figure 4I). Under serum‐free conditions, supplementing EGF induced phosphorylation of EGFR and its downstream signaling response within 60 min, which were significantly diminished in agrin‐silenced cells experiencing a stiff substrate (Figure 4J). Furthermore, sAgrin‐nourished stiff matrix markedly rescued EGF‐dependent activity of EGFR (Figure 4J). These observations suggest that the transmission of rigidity responses conveyed by agrin activates EGFR in both EGF‐dependent and‐independent manners in mutant cell types.

### 2.5 Agrin‐Dependent Matrix Mechanosensitizes EGFR to Integrins

**Figure 5:**
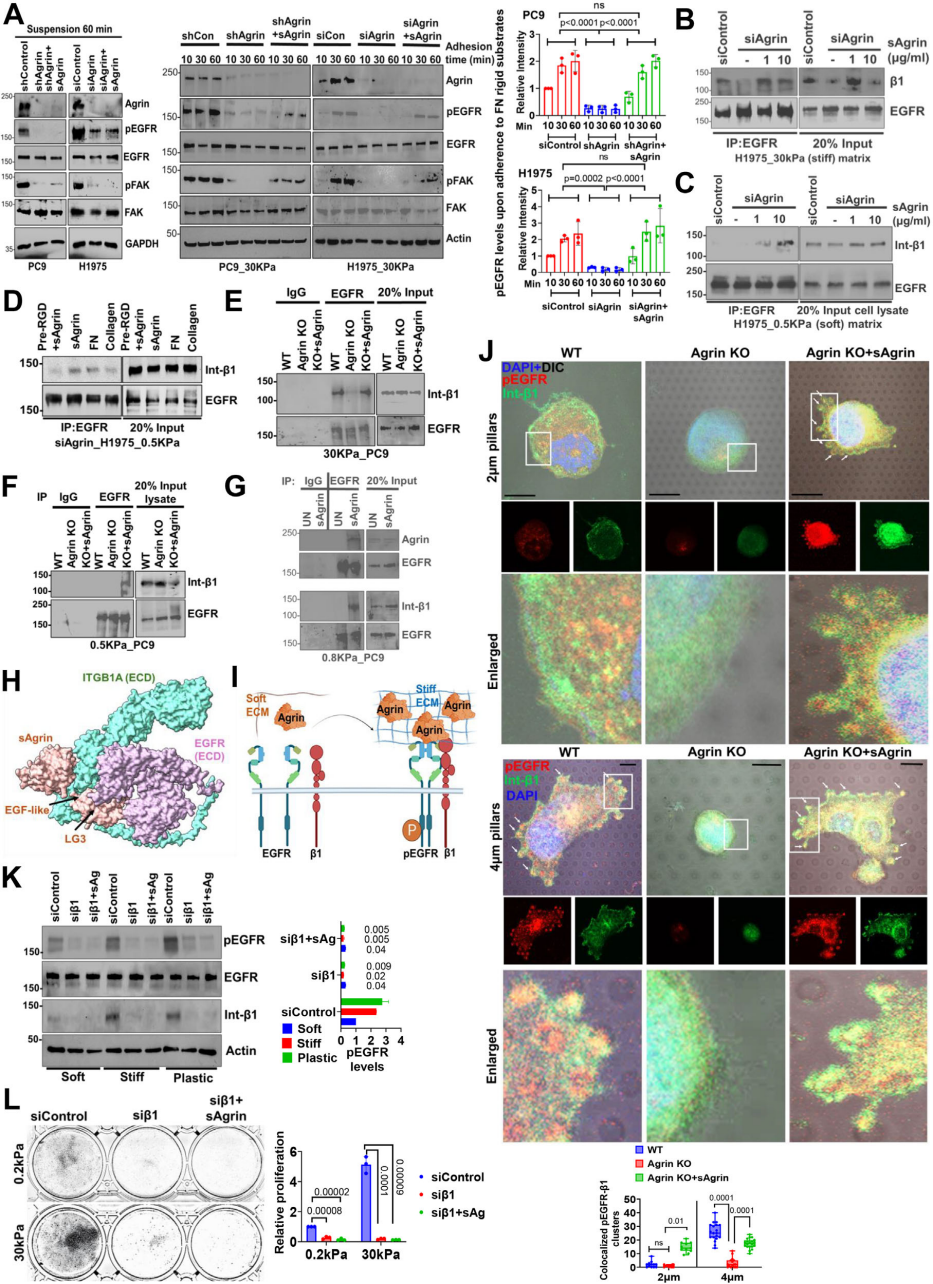
Agrin‐dependent matrix mechanosensitizes EGFR to integrin β1. A) Adhesion on indicated control, agrin‐depleted cells, and those pre‐treated with 10 µg/mL sAgrin for 18 h on 30 kPa. At indicated timepoints, cell lysates were analyzed by Western blotting for indicated proteins. β‐Actin served as the loading control. Data quantified as the mean pEGFR band intensity +/− SD, *n* = 3, Student's t test, *p* values indicated). Left panel shows Western blot from cells that were suspended (non‐adherent) for 1h. B–G) Co‐immunoprecipitation (Co‐IP) with EGFR antibody in control and agrin‐depleted cells on 30 kPa (B, E) or 0.5 kPa (C, D, F, and G) treated with indicated amounts of sAgrin for 18 h. For panel D, agrin knockdown cells were either pre‐treated with RGD inhibitor for 2 h before being treated with sAgrin, or sAgrin (10µg/mL), FN (10µg/mL) and collagen‐1 (10µg/mL) for 18 h, and subsequently the EGFR‐pulldown assay was performed. The blot was probed for integrin β1 and EGFR as the control. Twenty percent whole cell lysates were run as input (repeated thrice). H) Molecular docking surface model simulation showing the sAgrin‐induced (beige) juxtaposed with the extracellular domains (ECD) of EGFR (purple)‐Integrin β1 (cyan). I) A working model for agrin deposition in stiff ECM that clusters pEGFR to integrins. J) WT and agrin KO cells were plated on 2 or 4 µm pillars alone or containing 10 µg/mL sAgrin for 4–6 h. Representative confocal images stained for pEGFR and integrin β1 are shown. The number of co‐localized clusters presented as the mean +/− SD, *n* = 15–20 cells/condition, Student's t test, *p* values indicated. Scale bar: 10 µm. White arrows indicate clusters. (K) Western blot showing EGFR activation in control or β1‐silenced cells alone or treated with 10µg/mL sAgrin for 18 h on soft, stiff and plastic plates. Actin served as loading controls. The mean pEGFR band intensity +/− SD is presented, *n* = 3, Student's t test, *p* values indicated. L) Representative brightfield images of colony formation in H1975 on soft and stiff substrates from panel (I) (*n* = 3, multiple t test, *p* values indicated).

Retrospective to the crosstalk between clustered integrins with EGFR, pEGFR is known to localize to integrin‐focal adhesions in response to increased matrix rigidity.^[^ ^10^ ^]^ Therefore, we assessed if agrin mediated rigid ECM facilitates pEGFR activity during cell–substrate interactions via integrin engagement. First, we observed that adhesion to a fibronectin (FN), a ligand for integrin engagement‐coated rigid surface caused a rapid induction of EGFR phosphorylation within 10 min that was sustained throughout 60 min post‐adherence in control cells, which was significantly suppressed in agrin‐knockdown cells (**Figure** **5**A). Importantly, the failure to respond to the matrix due to agrin knockdown was rescued when sAgrin was added to the stiff matrix (Figure 5A). During such adhesion, agrin depletion also impaired focal adhesion activity, as reported in our previous studies^[^ ^13a^ ^]^ (Figure 5A). Importantly, sAgrin was not sufficient to rescue the loss of EGFR and FAK phosphorylation in agrin‐silenced cells in suspension (Figure 5A, left panel), indicating that adherence to agrin‐refurbished ECM is necessary for EGFR activation. Second, we tested integrin β1, as it is a well‐known co‐receptor for agrin in liver cancer cells.^[^ ^13a,e^ ^]^ To this end, we observed minimal interactions between agrin, integrin β1, and EGFR in compliant matrices (0.5 kPa) (Figure S5A, Supporting Information). However, consistent with the increase in agrin expression with matrix rigidity, tuning substrate stiffness (to 30 kPa) alone increased agrin–integrin β1 and EGFR complex formation (Figure S5A, Supporting Information). On stiff substrates, agrin depletion hampered the association of integrin β1 with EGFR and this was rescued in a dose‐dependent fashion upon the exogenous addition of sAgrin (Figure 5B). The interaction of integrin β1 and EGFR on soft substrates was not detected in control cells but was facilitated by sAgrin treatment on agrin‐silenced cells (Figure 5C). Of note, the sAgrin‐facilitated interaction of integrin β1 and EGFR on soft substrates in agrin‐knockdown cells was inhibited by pre‐treatment with RGD peptides that block integrin signaling (Figure 5D). In contrast, FN being a classical ligand for most integrins including β1, partly rescued this interaction, an effect which was not achieved by collagen treatment (Figure 5D). These results indicate that integrins present a critical link for sAgrin‐EGFR interactions. Likewise, agrin KO impaired the integrin β1–EGFR interactions in stiff matrix, which was rescued by sAgrin in both soft and stiff conditions (Figure 5E,F). Similar results were observed in agrin‐depleted cells, i.e., a lack of integrin β1–EGFR association at the leading edges that was rescued by sAgrin in stiff matrix (Figure S5B, Supporting Information). Remarkably, compliant matrix supplemented with supraphysiological levels of sAgrin promoted integrin β1–EGFR interactions, suggesting that increased agrin may mimic matrix rigidity responses and is a key driver in triggering EGFR‐integrin associations in stiff matrix (Figure 5G). Considering that sAgrin harbors EGF‐like and Laminin‐G3 (LG3) domains, we next simulated the tripartite molecular interaction involving the extracellular domains (ECD) of EGFR (amino acid residues 25–645) and integrin β1 (residues: 21–738) using Alphafold 3.0 predictions.^[^ ^27^ ^]^ Indeed, sAgrin appears as a molecular bridge stabilizing the interaction of EGFR and Integrin β1 as the LG3 domain of sAgrin makes potential contacts with both receptors (Figure 5H; Figure S5C, Supporting Information), though further biochemical and structural studies are needed to support this model. As a simplistic validation of the proposed model, we generated the ECD‐bearing versions of EGFR‐Fc‐tagged and integrin‐β1 (His‐tagged). Upon immobilizing Fc‐EGFR‐ECD to agarose beads, we observed that sAgrin treatment majorly favored the interaction between integrin β1 and EGFR (Figure S5D). Hence, this molecular bridging function mediated by sAgrin possibly clusters Integrin β1 to EGFR in mechanosensitive adhesion sites in response to increased agrin with tumor stiffness (Figure 5I).

### 2.6 Agrin Activates EGFR‐Integrin Mechanosignaling through Localized ECM‐Cell Contacts

After testing the role of agrin in bulk rigidity conferred through continuous cell‐ECM exposure to sensitize EGFR signaling, we further evaluated if microforce sensing through localized/discontinuous ECM‐cell mechanics as experienced by altering the adhesion size to substrates was similarly relayed by agrin to EGFR via integrins. To this end, we cultured lung carcinoma cell lines on polydimethylsiloxane (PDMS) pillars with diameters of 2 µm (that present a smaller adherent area and 6 μ height) and 4 µm (larger adhering area and 2 μ height) coated with fibronectin mimicking the “low or high mechanosensitive junctions” respectively. We estimated that cell attachment sites to small pillars (2.0 µm) would elicit less mechanical force (Spring constant ≈8.73 nN/µm) than those for cells attached to large pillars (4.0 µm diameter rendering a higher spring constant of ≈3770 nN/µm) (Figure S6A, Supporting Information). Both control and agrin KO cells remained circular with low confinement upon adherence to 2 µm pillars, indicative of their loss of contractility and spreading in these conditions accompanied by low cell‐elongation index (Figure S6B). Interestingly, these cells spread dramatically when the 2 µm pillars were coated with sAgrin, which significantly increased their elongation index (Figure S6B, Supporting Information) and led to efficient recruitment of pEGFR to these attachment sites in a dose‐dependent manner simulating EGFR activity mediated via agrin “high mechanojunctions” (Figure S6C, Supporting Information). Corroborating to a confined morphology, there was minimal pEGFR and integrin β1 in the sites adhering to the untreated 2 µm pillars, implying insufficient mechanoresponse (Figure 5J, top panels). Again, sAgrin‐coated 2 µm pillars significantly recruited pEGFR and integrin β1 (Figure 5J). In contrast, control cells sensing 4.0 µm diameter pillars had an increased elongation index that led to high pEGFR and integrin β1 colocalization in these attachment sites that was impaired upon agrin KO (Figure 5J; Figure S6B, Supporting Information). The 4 µm pillars coated with sAgrin significantly rescued the cell spreading and recruitment of activated EGFR (Figure 5J; Figure S6C, Supporting Information). Consistently, these results suggest that both continuous and localized mechanoresponses are orchestrated by agrin to sensitize EGFR by promoting interactions with integrin β1 (Figure 5I). Silencing integrin β1 abolished pEGFR levels on soft and stiff substrates which were not rescued by sAgrin (Figure 5K; Figure S6D, Supporting Information), resulting in the loss of proliferation and invasiveness (Figure 5L; Figure S6E, Supporting Information). Likewise, treatment with RGD peptides also reduced the stiffness‐induced EGFR‐phosphorylation and proliferative potential which was not rescued by sAgrin (Figure S6F–G, Supporting Information). Hence, these functional studies unmask the essentiality of integrin β1 for sustaining agrin‐EGFR‐induced oncogenic function.

### 2.7 Agrin‐EGFR Signaling Converges on YAP/TAZ

**Figure 6:**
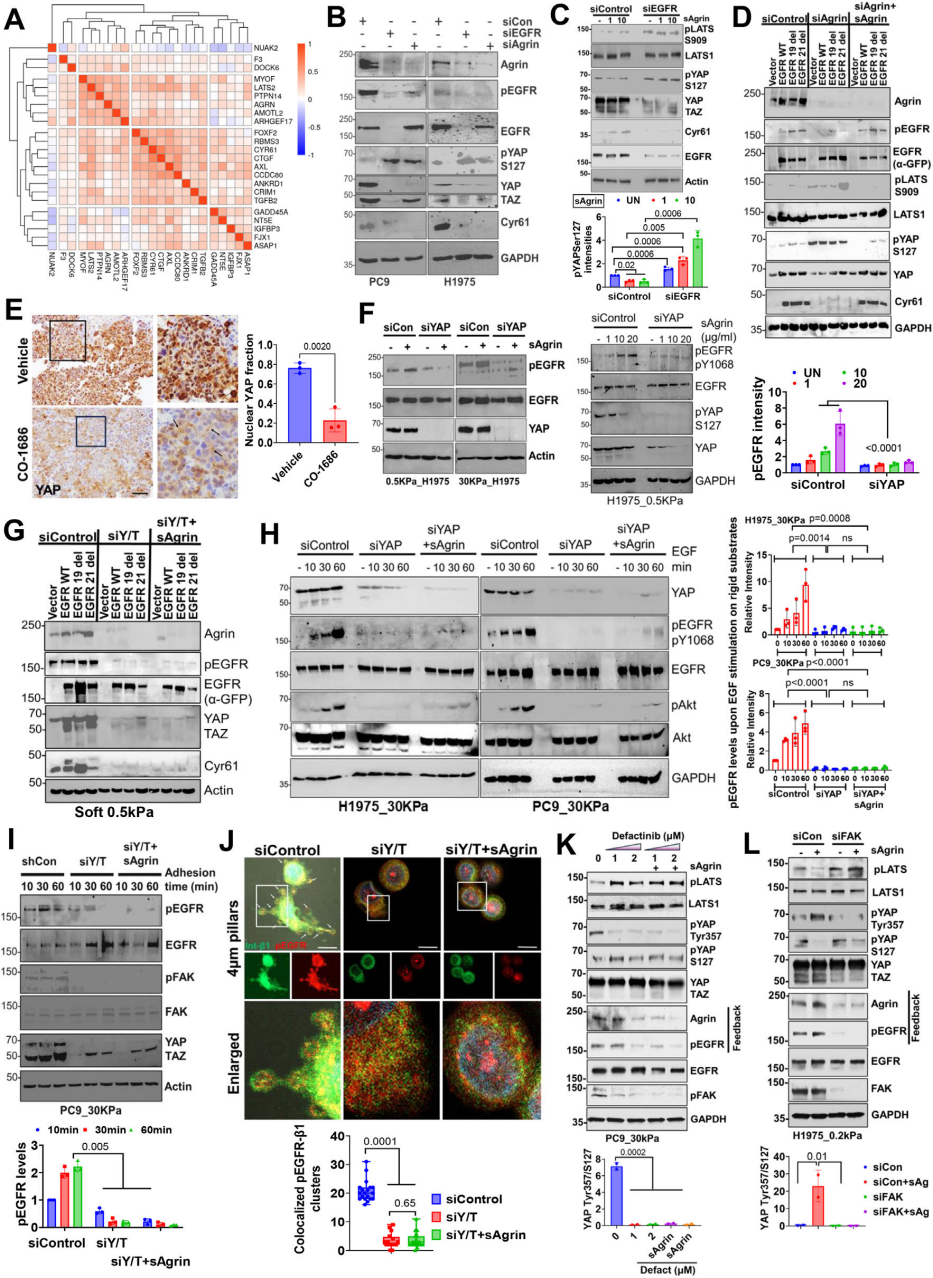
YAP/TAZ feedback on agrin–EGFR mechanotransduction. A) Heatmap showing AGRN amongst YAP/TAZ target genes based on TCGA_LUAD datasets (*n* = 515). B) Western blot analysis in the indicated control, EGFR, and agrin‐silenced cell lines for the indicated proteins. GAPDH served as the loading control (three biological repeats). C) Western blot analysis in whole‐cell lysates from control and EGFR knockdown H1975 treated with increasing concentrations of sAgrin for 18 h. β‐Actin served as the loading control. The mean pYAP± SD is shown (*n* = 3, multiple t tests). D) Indicated stably expressed H1299 cells expressing vector, EGFR‐WT, or its mutants were treated with control or agrin siRNA. After 72 h, one batch of agrin knockdown cells were treated with 10 µg/mL sAgrin for 18 h. Cell lysates were analyzed by Western blot for the indicated proteins. GAPDH served as the loading control (three biological repeats). E) IHC images of EGFR–L858R–T90 M mouse lung adenocarcinoma after vehicle or CO‐1686 treatment showing the YAP expression. Scale bar: 100 µm. Boxed region presents the enlarged panel. Black arrow shows cytoplasmic YAP. Nuclear YAP fraction represented as the mean +/− SD (*n* = 3 tumors per group, unpaired Student's t test, *p* value indicated). F) Western blot analysis of cell lysates from control or siYAP H1975 cells on 0.5 or 30 kPa in the presence/absence of 10 µg/mL sAgrin (left panels) or increasing doses (right panels) for 18 h on 0.5 kPa (right). β‐Actin served as the loading control (*n* = 3 repeats, data quantified as the mean pEGFR band intensity +/− SD, One‐Way ANOVA, *p* values indicated). (G) Western blot of H1299 cells expressing vector, EGFR‐WT, or its mutants were treated with control or YAP/TAZ (Y/T) siRNA and treated the same as in (D). (H) Western blot analysis of whole cell lysates from the control, siYAP, and those treated with sAgrin on 30 kPa substrates in the presence of EGF treatment for the indicated timepoints. GAPDH served as the loading control, *n* = 3 repeats, data quantified as the mean pEGFR band intensity +/− SD, Student's t test, *p* values indicated. I) Adhesion on the indicated control, Y/T‐depleted cells, and those pre‐treated with 10 µg/m sAgrin for 18 h on 30 kPa. At indicated timepoints, cell lysates were analyzed by Western blotting for indicated proteins. β‐Actin served as the loading control, *n* = 3, data quantified as the mean pEGFR band intensity +/− SD, Student's t test, *p* values shown. J) Control and Y/T‐depleted cells were plated on 4 µm pillars alone or containing 10 µg/mL sAgrin for 4–6 h. Representative confocal images stained for pEGFR and integrin β1 are shown. The number of co‐localized clusters presented as the mean +/− SD, *n* = 10–20 cells/condition, Student's t test, *p* values indicated. White arrows indicate clusters. Scale bar: 10 µm. (K) PC9 cells on 30 kPa were treated with indicated dose of defactinib for 4 h alone or in the presence of pre‐treatment with sAgrin for 16 h. Resulting cell lysates were analyzed for the indicated proteins. GAPDH served as a loading control (*n* = 2 repeats). (L) Control and FAK knockdown H1975 on 0.2 kPa were stimulated by sAgrin (10µg/mL) for 16h. Cell lysates were analyzed by Western blotting for the indicated proteins. GAPDH served as a loading control. Quantification of pTyr357/S127 of YAP is shown for panels K‐L (*n* = 2 repeats, multiple t tests).

YAP/TAZ are well‐defined mechanosensors that respond to tissue stiffness and other mechanical signals.^[^ ^13^, ^28^ ^]^ Because agrin relays such mechanical signals to YAP/TAZ via integrins in liver cancer cells,^[^ ^13e^ ^]^ the proposition of agrin‐dependent matrix sensitization of EGFR via YAP/TAZ activation becomes pertinent. Initially, we tested the clinical relevance of agrin–EGFR expression for YAP/TAZ activation in LUADs. An analysis of the single cell RNA transcriptomic profile of LUAD patients suggested that YAP/TAZ co‐distributed with agrin–EGFR expressions in the epithelial cells fraction of LUAD (Figure S7A, Supporting Information). Clustering with the well‐defined target genes for YAP/TAZ (cluster 3), revealed agrin as one of the key targets driven by YAP/TAZ that corresponded to a poor survival outcome (**Figure** **6**A). Through multivariable Cox regression involving YAP/TAZ genes (>100 genes), we identified several high‐risk YAP/TAZ regulated genes significantly associated with poor prognosis in LUAD, as indicated by their hazard ratios (HR) (Figure S7B, Supporting Information). Higher AGRN expression is notably associated with higher risk (HR = 1.25, *p* = 0.012). (Figure S7B, Supporting Information). DepMap analysis highlighted agrin as a co‐dependent gene for the YAP/TAZ–TEAD pathway, the loss of which negatively impacted cell proliferation (Figure S7C, Supporting Information). Principal component analysis (PCA) illustrated that agrin–EGFR expression along with other YAP/TAZ targets, aligned together in lung tumors distinct from the normal or tumor‐adjacent tissues (Figure S7C, Supporting Information). Consistent with the above, an inverse correlation of pEGFR and the inhibitory YAPS127 was observed in LUAD patient datasets that harbored EGFR mutations with high agrin (Figure S7D, Supporting Information). Similarly, LUAD patients with EGFR mutations revealed increased nuclear retention of YAP within lung tumors (Figure S7E, Supporting Information).

Knockdown of agrin or EGFR increased YAPSer127 phosphorylation, likely due to the activation of Hippo core kinases resulting in loss of expression of YAP target Cyr61 (Figure 6B; Figure S8A, Supporting Information). Because Merlin phosphorylation at Ser518 is known to inactivate its tumor suppressor function,^[^ ^29^ ^]^ this was reduced upon agrin silencing resulting in its activation coherent with our previous studies (Figure S8A, Supporting Information).^[^ ^13e^ ^]^ Consequently, agrin knockdown increased LATS1 (pS909) phosphorylation which was reduced by sAgrin treatment (Figure S8A, Supporting Information), further indicating that the activation of core‐Hippo kinase occurs in the absence of agrin. Thus, effects on Merlin and LATS phosphorylation events suggest that agrin‐EGFR signaling influences YAP/TAZ activity through the regulation of upstream Hippo pathway components. Interestingly, sAgrin did not rescue the loss of YAP activity in EGFR silenced cells, as evidenced by the increased LATS1/2 and YAPS127 phosphorylation and reduced Cyr61 expression (Figure 6C). Thus, agrin required EGFR to relay an activating response to YAP. The destabilization of YAP/TAZ observed upon EGFR silencing is consistent with the increased Ser127 that likely induces Ser381 phosphorylation by LATS1/2 which targets YAP for proteasomal degradation.^[^ ^28^, ^30^ ^]^ The expression of wild type or mutant versions of EGFR in H1299 cells enhanced agrin expression that accounted for increased YAP activity by lowering LATS1/2 phosphorylation that mediated YAPSer127 phosphorylation and subsequently increased Cyr61 expression (Figure 6D). Agrin silencing enhanced the inhibitory effects on YAP by increasing LATS1/2 mediated phosphorylation on YAP Ser127 phosphorylation, which decreased Cyr61 levels, an effect rescued by sAgrin treatment (Figure 6D). Consistent with the observed loss of agrin with EGFR inhibitor, a significant loss of nuclear YAP localization was noted in mouse lung tumors following CO‐1686 therapy (Figure 6E). These results were recapitulated in vitro where osimertinib similarly inhibited agrin–YAP activation (Figure S8B, Supporting Information). Overall, these findings highlight a mutual dependency between agrin and EGFR in YAP activation in lung cancer cell lines.

### 2.8 YAP/TAZ Feedback on Agrin‐EGFR Mechanosensing

The hypothesis that agrin–EGFR mechanotransduction converges on YAP/TAZ posited us to examine if YAP/TAZ sustains this mechanical module by promoting agrin and/or EGFR expression through a mechanotransductive oncogenic feedback loop. Overexpressing YAP or its constitutively active mutant YAP5SA‐increased agrin mRNA and protein levels (Figure S8C,D, Supporting Information). While no changes in EGFR levels were observed, although its phosphorylation status was upregulated upon YAP or YAP5SA expression, likely due to the increase in agrin protein levels (Figure S8C,D, Supporting Information). In contrast, YAP knockdown was sufficient to reduce agrin mRNA and protein levels (Figure S8E, Supporting Information). Consistently, the sAgrin‐induced pEGFR activity observed in soft matrix was markedly suppressed by YAP silencing (Figure 6F). The increased expression of agrin induced by overexpressing EGFR or its mutants was suppressed by depletion of YAP/TAZ resulting in reduced EGFR activity and loss of YAP‐dependent transcription as indicated by Cyr61 expression (Figure 6G). Similar results were achieved when TEAD4, the transcription factor utilized by YAP/TAZ to drive target gene expression, was silenced (Figure S8F, Supporting Information). Treatment with VT107, a potent TEAD auto‐palmitoylation inhibitor,^[^ ^31^ ^]^ blocked agrin as a target gene of YAP/TAZ‐TEAD and EGFR activity in a dose‐dependent manner in addition to YAP/TAZ inactivation and reduced expression of Cyr61 (Figure S8G, Supporting Information). These results imply that agrin expression in the corrupted lung ECM is majorly driven by YAP/TAZ–TEAD and the TME with increased agrin also sensitizes EGFR and YAP activation.

Several lines of evidence further suggest that agrin was unable to relay matrix rigidity responses to activate EGFR signaling in the absence of YAP/TAZ. First, YAP/TAZ depletion reduced EGF‐induced pY1068 phosphorylation in lung cancer cells experiencing a stiff matrix, which was not rescued by sAgrin supplemented matrix (Figure 6H). Second, adhesion of PC9 cells to stiff matrix that initiated EGFR activity was markedly abrogated in YAP/TAZ‐depleted conditions to an extent that could not be restored by sAgrin in the stiff matrix (Figure 6I). Consistent with a loss of spheroid growth and protrusive structures (Figure S8H, Supporting Information), YAP/TAZ depletion impaired the activation of Focal Adhesion Kinase (FAK), which was not rescued by sAgrin (Figure 6I), thus suggesting that YAP/TAZ is necessary for the adherence and proliferation of lung cancer cells on stiff matrix. Consistent with the regulation of agrin by YAP/TAZ‐TEAD, we observed robust occupancy of YAP1, TEAD1, and TEAD4 in the second intron of AGRN gene from CistromeDB analysis, suggesting the existence of enhancer in response to YAP‐TEAD activation in AGRN gene, although the functional validation is yet to be proven (Figure S8I, Supporting Information, left panels). Notably, silencing YAP/TAZ and TEAD modestly decreased agrin and canonical YAP targets CTGF and CYR61 in soft matrix (Figure S8I, Supporting Information, right panel). In contrast, the increased expression of agrin and YAP/TAZ targets caused by matrix stiffness was drastically inhibited by both YAP/TAZ and TEAD knockdowns (Figure S8I, Supporting Information, right panel). The mechanosensitive complex formation between EGFR and integrin β1 facilitated by agrin in a rigid matrix was strikingly reduced in YAP/TAZ‐silenced cells (Figure S8J, Supporting Information). Moreover, the localized and bulk rigidity‐sensed colocalization between pEGFR and integrin β1 observed when cells attached to 4.0 µm pillars was attenuated by YAP/TAZ silencing (Figure 6J; Figure S8K, Supporting Information). In these settings, sAgrin‐coated 4.0 µm pillars were unable to sensitize EGFR–integrin β1 complexes (Figure 6J; Figure S8K, Supporting Information). Together, these results suggest that YAP/TAZ‐dependent agrin expression drives and sustains EGFR mechanoactivity toward localized and bulk rigidity.

To test the possibility that agrin‐induced FAK downstream of EGFR‐integrin mechanosensitive complex may phosphorylate YAP at Tyr357 to activate the latter,^[^ ^32^ ^]^ we treated PC9 cells on 30 kPa with an increasing dosage of a FAK inhibitor, defactinib, in the presence or absence of sAgrin. Defactinib treatment caused an increased LATS1/2 phosphorylation that resulted in enhanced YAPS127 phosphorylation but strikingly reduced Tyr357 phosphorylation of YAP (Figure 6K). Thus, reduced YAP activity as an outcome of decreased Tyr357 phosphorylation caused by defactinib was associated with lower agrin and EGFR phosphorylation which was not rescued by sAgrin (Figure 6K). These results were recapitulated by silencing FAK. The sAgrin treated control cells cultured on soft matrix decreased LATS1/2 and YAPS127 phosphorylation levels coupled to enhanced Tyr357 YAP phosphorylation, which was essentially reversed by FAK knockdown (Figure 6L). Likewise, FAK knockdown lowered agrin and EGFR phosphorylation displaying impaired YAP/TAZ feedback (Figure 6L). Further analysis showed that inhibiting actomyosin (by blebbistatin) that lowered FAK activation or PI3‐K (by LY294002) which impacted PI3‐K‐Akt activity, strongly reduced the sAgrin induced YAPTyr357 phosphorylation on 0.2 kPa resulting in increased YAPSer127 phosphorylation (Figure S9A,B, Supporting Information). Collectively, actomyosin and PI3‐K inhibition were associated with lower agrin and EGFR activity as an outcome of YAP/TAZ inactivation (Figure S9A,B, Supporting Information). In contrast, despite increased YAPSer127 phosphorylation caused by c‐RAF‐MAPK inhibition, GW5074 treatment did not decrease sAgrin‐induced Tyr357 activity of YAP, which had little impact on EGFR activity and agrin status on compliant matrices (Figure S9C, Supporting Information). These observations further demonstrate that FAK‐actomyosin coordinates with PI3‐K downstream of agrin‐EGFR mechanotransduction to trigger YAP/TAZ oncogenic feedback and that c‐RAF‐MAPK activity was dispensable for agrin‐EGFR mechanotransduction on YAP/TAZ (Figure S9D, Supporting Information).

### 2.9 Co‐Targeting EGFR‐YAP/TEAD Restricts Lung Tumorigenesis by Agrin Inhibition

**Figure 7:**
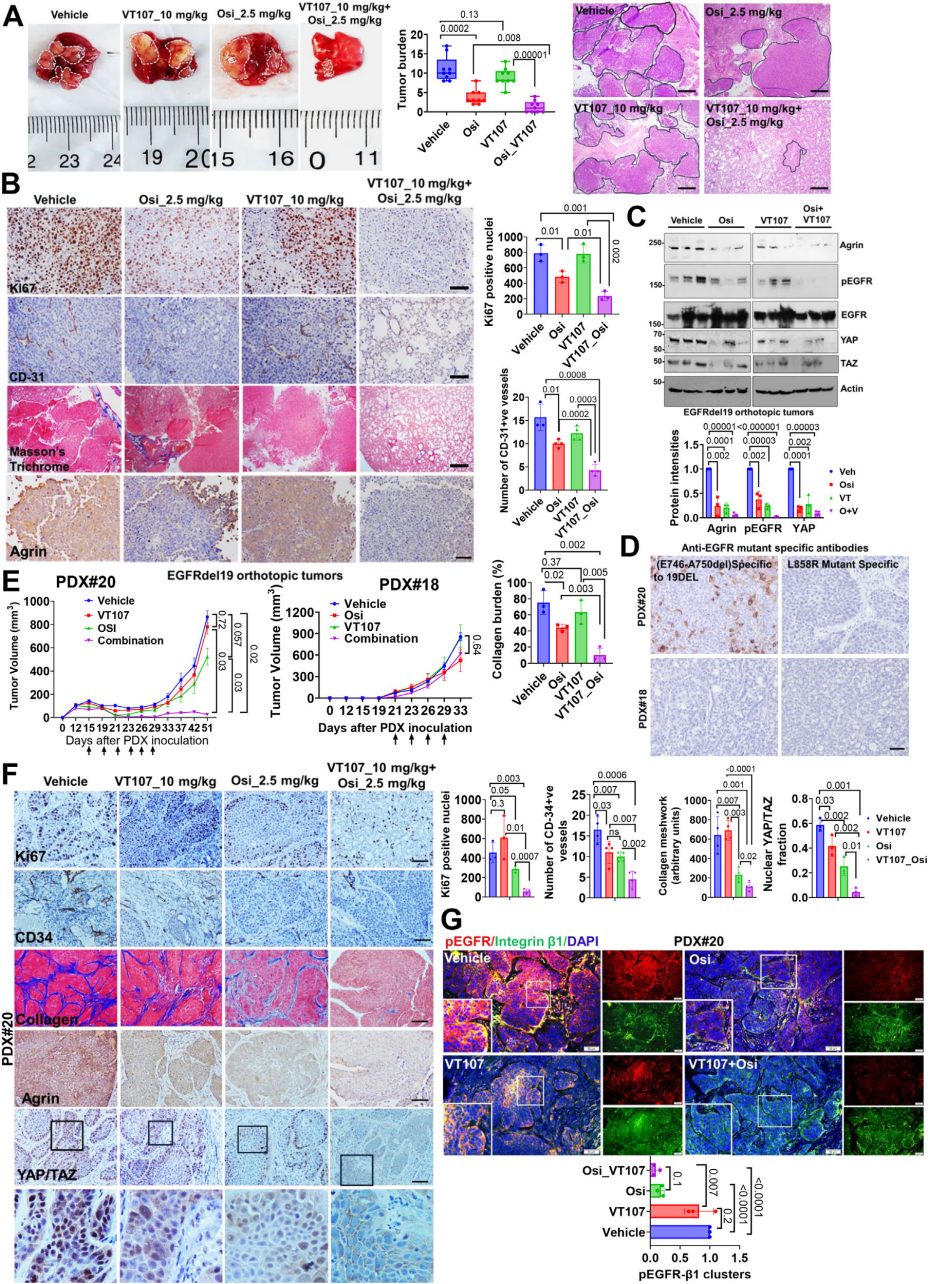
Dual targeting of EGFR–YAP/TEAD restricts lung tumorigenesis by agrin downregulation. A) Representative photograph of mouse lungs bearing tumors (marked by white dashed lines) derived from implantation of H1299del19 that received vehicle, osimertinib (2.5 mg kg^−1^), VT107 (10 mg kg^−1^), and their combinations for five times every 3 days. Scale: 1 cm. Hematoxylin and Eosin‐stained images of lungs from different treatments after one month. Scale bar: 50 µm. The number of visible tumor nodules (in black) are presented as the mean +/− SD (*n* = 9 mice/group, multiple t tests, *p* values indicated). B) Representative IHC images of mouse lung tumors for Ki67, CD‐31, and Masson's Trichrome amongst treatment groups as in (A). Scale bar: 50 µm. The mean intensities +/− SD are quantified (*n* = 3 tumors from three mice/condition, multiple t tests, *p* values indicated). C) Western blot analysis from lysates from lung tumors of different treatment groups for the indicated proteins. β‐Actin served as the loading control. Data quantified from *n* = 3 tumors from three mice, multiple t test, *p* values indicated). D) Representative IHC images for PDX#20 and #18 tumors stained with indicated EGFR‐mutant specific antibodies. Scale bar: 50 µm. (E) Tumor volumes of PDX#20 and #18 injected subcutaneously in NOD/SCID mice and receiving the vehicle, osimertinib (2.5 mg kg^−1^), VT107 (10mg kg^−1^), and their combination on indicated days (black arrow) (*n* = 5 mice/group). F) Representative IHC images of PDX#20 for Ki67, CD‐31, and Masson's Trichrome amongst various treatment groups as in (E). Boxed region of YAP/TAZ staining presented as enlarged panels. Results are presented as the mean +/− SD (*n* = 3–4 sections. from three mice per condition, unpaired Student's t test, *p* values indicated). Scale bar: 100 µm. G) Confocal microscopy images showing pEGFR and integrin β1 mechanosensitive junctions in PDX#20 tumors treated as in (E). Scale bar: 50 µm. Inset shows the enlarged region. The co‐localized EGFR‐β1 clusters presented as the mean +/− SD (*n* = 3 sections from three tumors, Student's t test, *p* values indicated).

Since YAP/TAZ–TEAD and EGFR represent a major dependency in agrin‐mediated cell proliferation (Figure S10A, Supporting Information), we examined whether combined inhibition of EGFR and YAP/TAZ transcriptional activity reduced the oncogenesis through agrin impairment. As an upstream activator of EGFR, silencing agrin suppressed the growth of EGFR‐mutant cell lines which was further amplified with Osimertinib treatment (Figure S10B, Supporting Information). Likewise, the increased proliferation attributed to WT or mutant EGFR was, in part, suppressed by agrin or YAP/TAZ silencing (Figure S10C). Although overexpressing EGFR or its mutant could also promote growth through agrin‐YAP independent pathway which could account for a partial impact of agrin or YAP silencing. Compared to each inhibitor alone, combination of osimertinib and VT107 showed a lower IC50 for inhibiting cell proliferation (≈4.5 and 207 nM in H1975 and PC9, respectively) in control cells, which was further reduced in agrin‐depleted cells (3.9 nM, H1975; ≈112 nM, PC9) (Figure S10D, Supporting Information). Consistently, the combination of osimertinib and VT107 exerted strong anti‐proliferative effects in clonogenic assays and substantially reduced agrin, pEGFR, and YAP/TAZ, as well as CTGF as a well‐defined target (Figure S10E,F, Supporting Information). This observation prompted us to test whether the combination of osimertinib and VT107 inhibited lung tumorigenesis by impairing agrin–EGFR–YAP signaling. First, we orthotopically implanted H1299–EGFRdel19‐GFP expressing cells in SCID mouse lungs to mimic the development of the EGFR–del19 genetically engineered mouse model (GEMM). After 12 days following the confirmation of tumor growth by magnetic resonance imaging (MRI), we began treatments with a modest dosage of VT107 (10 mg kg^−1^) and osimertinib (2.5 mg kg^−1^) as monotherapies, and their combinations for five times every 3 days. At one‐month post‐implantation, vehicle‐treated groups developed robust tumor nodules reminiscent of LUAD (**Figure** **7**A). As monotherapies, osimertinib significantly reduced the tumor burden while VT107 had negligible effects (Figure 7A). Remarkably, VT107 potentiated the effects of osimertinib by drastically alleviating the tumor burden in the mice that received the combination therapy (Figure 7A). This was evidenced by reduced proliferation and normalization of blood vessels as shown by Ki67 and CD‐31 staining, respectively (Figure 7B). While the vehicle, VT107, and Osimertinib treated groups had strong tumor‐associated collagen deposition, the combination treatment significantly restored collagen distribution as expected within normal lungs (Figure 7B). Analysis of the extracted lung tumor tissues revealed the lowest agrin expression in the combination group when compared with the vehicle, osimertinib, and VT107 treated ones, which was consistent with the loss of EGFR phosphorylation, reduced YAP stability and nuclear localization (Figure 7C; Figure S10G, Supporting Information).

Lastly, we examined 20 human lung cancer patient‐derived xenografts (PDXs) for agrin, EGFR, and YAP expression. Several PDX tumors expressed these targets (Figure S10H, Supporting Information). Notably, we revived the growth of two adenocarcinomas—PDX#18 (with low agrin–YAP and no evidence of EGFR mutation) and PDX#20 (high agrin, moderate EGFR with del19 mutation, and high YAP) in SCID mice used for validating the efficacy of osimertinib–VT107 combinations (Figure 7D; Figure S10H, Supporting Information). Consistent with our orthotopic model, VT107 did not significantly inhibit PDX#20 growth, whereas osimertinib treatment resulted in modest tumor regression that was not statistically significant (Figure 7E, left panel). Interestingly, the combination of VT107 with osimertinib significantly diminished PDX#20 growth (Figure 7E). In contrast, the combination therapy showed little effect on the tumor volume of PDX#18 as these tumors had low EGFR–YAP as drug targets, which did not impact agrin (Figure 7E, right panel). The reduction in proliferation, blood vessel number, tumor‐fenestrated collagen, and nuclear YAP/TAZ was consistent with agrin loss in PDX#20 in response to the combination therapy, while no major differences amongst the non‐responsive PDX#18 tumors were observed (Figure 7F; Figure S10I, Supporting Information). The combination drug strategy did not appear to be toxic in mice bearing PDX#20 (Figure S10J, Supporting Information). Consistent with the in vitro findings that agrin regulates EGFR–integrin β1 associations, integrin β1 marked cell surface regions were enriched with active EGFR amongst the vehicle‐treated PDX#20 tumors, which were slightly impacted by VT107 but to a larger extent by osimertinib (Figure 7G). Strikingly, the combination therapy abolished these pEGFR enriched integrin β1 mechanosensitive regions which was consistent with agrin loss (Figure 7G). Collectively, these in vivo results suggest that combined inhibition of EGFR and YAP/TEAD transcriptional activity diminishes lung tumorigenesis by limiting agrin expression, and therefore, reinforces the therapeutic value of restraining the positive feedback loop imposed by the agrin–EGFR–YAP/TEAD oncogenic axis that is sensitive to ECM rigidity. Our findings have significant implications for personalized therapeutic approaches in lung cancer. The identification of agrin as a key mediator of EGFR activation in response to matrix stiffness and its integration with YAP/TAZ TEAD suggests that patients with high agrin expression may benefit most from combination therapies targeting both EGFR and YAP/TAZ. Therefore, we anticipate that clinical studies should investigate agrin expression as a potential biomarker for predicting response to such combination treatments, particularly in patients with EGFR mutations.

## 3 Discussion

The cancer‐refurbished ECM plays a vital role in assembling key growth factor receptors to engage discrete mechanosignaling within the cancer cells, which in turn revamps the ECM.^[^ ^12a^ ^]^ Central to this bidirectional communication are the integrin‐mechanosensing pathways and the timely engagement of RTKs such as EGFR. While the stiff pro‐tumorigenic ECM activates EGFR likely through integrin‐focal adhesion,^[^ ^6^, ^8^, ^9^, ^10^, ^33^ ^]^ the ECM components aiding these processes have been largely undefined. In this context, several new insights from this study unmask agrin as a mechanoinducer of EGFR in the stiff lung TME and provide a molecular picture of the mechanosignaling initiated by agrin in the ECM, crosstalk at the cell–ECM interface with EGFR, and transmitting signals to nuclear YAP/TAZ for sustaining agrin levels via positive feedback loop. This agrin‐EGFR‐integrin‐YAP/TAZ‐agrin positive cycle may be of great relevance for the stiff lung cancer matrix in patients harboring EGFR mutations. First, consistent with the clinical observation that agrin is expressed in EGFR‐amplified tumors, EGFR controls the tumorigenic agrin expression in vitro and in multiple EGFR‐driven genetic mouse LUAD models. Second, the physiological impact and molecular basis of EGFR‐induced tumorigenic agrin expression via EGFR‐integrin‐YAP/TAZ were revealed in our work. As an unexpected mechanism, agrin provided ECM‐stiffness cues that activated both wild‐type and mutant EGFR, sustaining its proliferative capabilities in cancer cells. Third, agrin serves as a non‐canonical ligand that conveys tissue rigidity signals to EGFR and thereby links it to integrin mechanosignaling. Fourth, agrin–EGFR mechanotransduction converges on the YAP/TEAD pathway through the FAK‐acto‐myosin‐PI3‐K activation that further drives agrin expression as a feedback response to sustains this oncogenic module. Finally, targeting the EGFR–YAP/TEAD mechanosignaling module reduced lung tumorigenesis in association with the depletion of agrin in orthotopic mouse models and those bearing human LUAD PDX. This represents an innovative mechanism of action (MOA) for existing therapies against mutant EGFR and TEAD especially for tumors having high agrin expression, which may serve as a predictive biomarker.

The clustering of RTKs such as EGFR has been shown to amplify ligand induced RTK signaling.^[^ ^9^, ^34^ ^]^ For EGFR, this mainly occurs via receptor homodimerization upon EGF/other canonical ligand binding. Our results imply that agrin‐enriched bulk and local rigidity serve as “mechanical recognition” sites empowering EGFR homodimerization and unleashing downstream signal amplification in the TME. This agrin‐induced EGFR amplification occurred both in the presence or absence of EGF. In contrast, EGF alone was insufficient to rescue EGFR phosphorylation upon agrin silencing in stiff matrix but was able to rescue it when combined with sAgrin. This evidence suggests that increased agrin cues, resulting from the formation of a stiff matrix, are necessary for both EGF‐induced or EGF‐independent EGFR phosphorylation. Consistent with the emerging notion that the ECM serves as a “molecular sink” that harbors growth factors capable of activating RTKs such as EGFR, our evidence additionally indicates that interaction of agrin with the ECM may itself trigger mechanoactivation of EGFR coupling it to integrin β1 mechanosignaling. Likewise, agrin activated EphrinB2 in the erythroid niche that involved integrin β1.^[^ ^35^ ^]^ Advancing previous observations of EGFR activation within integrin‐focal adhesion clusters,^[^ ^10^ ^]^ our study suggests that agrin integrates EGFR signals to integrin‐focal adhesions during both localized adherence and bulk rigidity experiencing interfaces of cancer cells with their ECM.

YAP/TAZ forms the roots of mechanosignaling associated with tumorigenesis and organ development.^[^ ^36^ ^]^ Despite the evidence that YAP/TAZ mediate lapatinib resistance in stiff tumor environments for HER2‐amplified cancers,^[^ ^37^ ^]^ not much is known as to how these signals are conveyed via EGFR from the stiff ECM and their relevance to tumorigenesis. Our work directly addressed this issue by identifying a functional role for agrin in the stiff ECM, which activated EGFR in the presence/absence of EGF and linked these tissue rigidity responses to YAP–TEAD transcription. The observed feedback by YAP/TAZ, which replenished agrin by TEAD‐mediated transcription in the lung TME, was necessary to sustain the agrin–EGFR–YAP mechanosignaling. Our findings that illustrate the importance of FAK mediated Tyr357 activation of YAP are consistent with the recent studies that highlight EGFR‐FAK‐YAP/TAZ regulation as a mechanism of cancer resistance through nullifying the Hippo kinases.^[^ ^32^, ^38^ ^]^ Considering that long‐term treatments with osimertinib lead to the development of resistance amongst NSCLC patients through YAP‐mediated oncogenic bypass as one major pathway,^[^ ^39^ ^]^ the identification of agrin as mechanosensor for EGFR as well as YAP/TAZ target gene in sustaining tumor mechanotranduction is of significance. Driving these therapeutic advances is our proof‐of‐principle evidence showing that the potential of osimertinib can be maximized by combining it with TEAD inhibitor that restricts the tumorigenic agrin–EGFR–YAP/TEAD feedback. In this regimen, we employed suboptimal concentrations of both osimertinib and VT107 to maximize inhibition of agrin, reduce nuclear YAP/TAZ, and thereby limit lung tumorigenesis. A suboptimal dose for VT107 (10 mg kg^−1^) was not sufficient to completely reduce agrin levels; however, when used with osimertinib, the combination strikingly reduced agrin, which was associated with a lower tumor burden. Moreover, the combination therapy robustly expelled YAP/TAZ outside the nucleus thereby impacting its association with TEAD transcription, illustrating the importance of agrin–EGFR feedback in potentiating nuclear YAP/TAZ in lung cancers. VT107 strongly potentiated the impact of osimertinib in alleviating lung tumorigenesis, and therefore, underscoring that dual blockage of the EGFR–YAP/TEAD pathway is essential to limit agrin in the lung TME. Corroborating to the clinical observation that EGFR positive tumors are enriched with agrin–YAP, the combination treatments provide a solid platform to target EGFR mutant/amplified lung tumors and potentially limit subsequent resistance to therapies.

This study unravels several avenues for exciting future research. First, the role of agrin in mediating resistance to EGFR‐targeted therapies should be investigated, as high agrin expression may contribute to treatment resistance. Second, the development of agrin‐specific inhibitors or antibodies or RNAi approach could provide a novel therapeutic approach, potentially in combination with existing EGFR and YAP/TEAD inhibitors. Third, the broader applicability of the agrin‐EGFR‐YAP/TAZ axis in other cancer types, particularly those with high EGFR expression or activation, warrants exploration. Fourth, the potential of agrin as a predictive biomarker for combination therapies targeting EGFR and YAP/TEAD should be validated in larger clinical cohorts.

In conclusion, we reveal that agrin serves as a mechanosensing ECM protein for EGFR toward tumor stiffness. Agrin–EGFR mechanorigidity converges on YAP/TEAD transcription that sustains this mechanosignaling through feedback transcription of agrin. Importantly, combination treatments targeting EGFR and YAP/TAZ‐TEAD were effective in restraining lung tumorigenesis by blocking agrin as a central player in this oncogenic module, thereby revealing new vistas to combat EGFR‐driven lung malignancies. Notably, the expression of agrin may serve as a biomarker for predicting the outcome of targeted therapies directed against both EGFR mutations and YAP/TAZ‐TEAD transcriptional output.

## 4 Experimental Section

### Study Design

The objective of this study was to examine mechanosensitizing the role of extracellular matrix proteoglycan agrin on EGFR‐addicted lung adenocarcinoma. Employing a comprehensive range of in‐vitro and in‐vivo experiments, it was concluded that agrin serves as a mechanosensing ECM protein for EGFR toward tumor stiffness. EGFR‐driven mouse models show preferential increased agrin expression and this converges on the YAP/TAZ pathway to create a feedback oncogenic loop promoting tumorigenesis. Further studies using othotopic and human PDX models showed the efficacy of combined targeting of EGFR‐TEAD pathway in limiting agrin‐based lung adenocarcinoma development. The human lung cancer specimens were analyzed in a retrospective manner from the respective institutions, as approved by the Institutional Review Board of Roswell Park (protocol #166 123). Informed consent statement: Written informed consents were obtained and approved at the time of sample collection by the Data Bank and BioRepository Shared Resource (DBBR), Roswell Park Comprehensive Cancer Center under the BDR protocol #166 123. The number of experimental replicates is indicated in each panel of figure legend. All in vivo experiments were performed following the guidance of the Institutional Animal Care and Use Committee. Blinding was not used in this study. The health status of each mouse was monitored daily, and mice that met the study endpoints were humanely euthanized. For most assays, at least two‐three independent experiments were performed with three biological replicates.

### Cell Lines, Plasmids, and Genetically Edited Cells

NCI‐H1299 (EGFR WT), NCI‐H1666 (EGFR WT), NCI‐H460 (EGFR WT), NCI‐H1650, and HCC4006 (EGFR exon 19 deletion mutants), as well as A549 cell lines and primary immortalized human bronchial epithelial cells (HBLE) were obtained from the American Type Culture Collection (ATCC). The cells were maintained in RPMI, DMEM medium, or human bronchial epithelial cell‐based medium containing 10% fetal bovine serum (FBS). The PC9 cells expressing the EGFR exon 19 deletion mutation (E746‐750) were provided by Kazuto Nishio (National Cancer Center Hospital, Tokyo) and maintained in RPMI containing 10% FBS. Stable cells were generated by using NCI‐H1299 (EGFR WT) cells transfected with EGFR WT‐GFP, EGFR 19del‐GFP, or EGFR L858R‐GFP by application of Amaxa Kit C, and cells were selected with G418 at 500 µg/mL and sorted for green fluorescent protein (GFP) via flow cytometry as previously published.^[^ ^20^ ^]^ Agrin knock‐out in PC9 is achieved with the saCas9 system based on the PX602 plasmid.^[^ ^40^ ^]^ The bGH polyA with a shorter synthetic polyA signal was replaced to create space for a second sgRNA to enhance KO efficacy. Advantage of the web‐based sgRNA designer from the Broad Institute, MIT, was taken to design several Agrin‐specific sgRNAs candidates. The KO efficiency was determined by Western‐blot analysis. Lentiviral shRNA mediated Agrin knockdown in H1299‐Vector, wildtype and mutant EGFR expressing cells were performed using previously established protocols.^[^ ^13a,e^ ^]^ Lentiviral particles were packaged in human embryonic kidney 293T cells that were cotransfected with lentiviral vector and helper plasmids (psPAX2 and pMD2.G encoding Gag/Pol and vesicular stomatitis virus glycoprotein, respectively). Transduction was performed in the presence of 5 µg/mL polybrene (MilliporeSigma) as previously described.^[^ ^13a,e^ ^]^ The sequences of Agrin shRNAs are provided in Table S2 (Supporting Information). All cell lines underwent authentication to confirm their identity and were tested free of mycoplasma contamination (Lonza, LT07‐318).

### Mice and Tumor Models—CCSP–rtTA–EGFRT790M/L858R Transgenic Model

EGFR–L858R–T790 M transgenic mice were mated to CCSP–rtTA transgenic mice^[^ ^41^ ^]^ (Jackson Laboratories, RRID: IMSR_JAX:0 06232) to create double transgenic doxycycline‐inducible mice. Mice were given 2 g/kg doxycycline feed (Bio‐Serv) starting at 5 weeks of age. Tumor development was monitored by magnetic resonance imaging (MRI), and only mice with tumors confirmed by MRI were included in this study. Nodules were visible by 14–20 weeks post‐induction. Equal number of males/females were analyzed. The hEGFR‐L858R/T790 M knock‐in model into Collagen1a1 (Cola1) locus was obtained from Dr. Christine Brainson, University of Kentucky. The resulting mice had one Cola1 locus replaced by Lox‐Stop‐Lox (LSL) which was subjected to intranasal adenovirus‐Cre recombinase that induced lung tumors within 107–120 days post‐inhalation.^[^ ^19^ ^]^ The hEGFR‐T790M/L858R^LSL^ mice were obtained from Dr. David Goodrich, Roswell Park.

#### Mice and Tumor Models—Orthotopic Model

Female Severe Combined ImmunoDeficient (SCID) mice (6–8 weeks old female, purchased from the Division of Laboratory Animal Resources‐LAR, Roswell Park Comprehensive Cancer Center) were processed based on non‐surgical method of cancer cell implantation (See supplementary methods for additional details). After 15 days post‐implantation, one batch of mice were imaged by MRI to monitor tumor development. When tumor nodules >100 mm^3^ were visible, mice were randomly divided into four groups that received the vehicle (1% corn oil in phosphate buffered saline, PBS), osimertinib (2.5 mg kg^−1^ in 1% corn oil), VT107 (10 mg kg^−1^ in 1% corn oil), and the respective combinations for five injections every 2–3 days intraperitoneally (Mon., Wed., and Fri.).

#### Mice and Tumor Models—Lung Adenocarcinoma PDX Model

The experimental human lung PDX tumor animal model was established according to IACUC‐approved animal protocols. Briefly, all the female SCID mice were anesthetized using isoflurane. Lung PDX tumors maintained on SCID mice were isolated, and 30–40 mg of non‐necrotic tumor tissue was subcutaneously transplanted into the flank region of SCID mice. Macaine was used immediately after tumor tissue transplantation to relieve pain. When human tumors grew to 100–150 mm^3^, mice were randomly divided into the required groups for drug treatment. After the completion of the in vivo study, no signs of sickness, weight loss, or morbidity were observed during the treatment course. To evaluate potential side effects and toxicity of VT107, Osimertinib, or their combination, cytotoxicity assessments were conducted using the Trypan blue exclusion assay. Following the experiment's conclusion, bone marrow samples were collected from randomly selected mice in each group. The bone marrow cells were counted using the Beckman Vi‐CELL XR Cell Viability Analyzer. The results indicated no significant difference in viable cells between the treatment and control groups. All animal experiments performed were done in accordance with experimental protocols reviewed and approved by Division of Laboratory Animal Resources‐LAR, Roswell Park Comprehensive Cancer Center under strict compliance to the Institutional Animal Care and Use Committee (IACUC) guidelines for the ethical use of animal models in biological research (protocol #1489), Roswell Park Comprehensive Cancer Center.

### Substrate Stiffness Manipulation In Vitro and In Vivo

Tissue culture plates were coated with Col‐T‐gel (Fischer Scientific) with a stiffness ranging from 0.8 (soft) to 30 kPa (stiff) as per manufacturer recommendations, and these were allowed to solidify for 40 min at 37 °C inside a tissue culture incubator. About 0.8 kPa gel (Cat# P720S‐component a) or 30 kPa (Cat# P720H‐component a) with component b (supplied by the company) were mixed to obtain the desired stiffness following manufacturer's recommended guidelines. The mixture was layered on the tissue culture plates before adding the cells. Other experiments were performed with cells seeded on polyhydrogel plates of the following defined stiffness: 0.2, 0.5, or 0.8 kPa (soft) and 30 kPa (stiff) silicone polyhydrogels (CytoSoft, Advanced Biomatrix, Inc.). The determination of stiffness of silicone substrates was done by the manufacturer as per previously established protocols.^[^ ^12^, ^42^ ^]^ For in vivo studies, 2.5 × 10^6^ cancer cells in 50 µl complete media were mixed with 50 µl of 1:1 VitroGel RGD High Concentration (The Well Bioscience) (gel:dilution buffer) to generate a stiff microenvironment corresponding to ≈10 kPa. For soft conditions, the same number of cells were mixed with 50 µl of 1:5 VitroGel RGD, which corresponds to 0.5 kPa as per the manufacturer reported quality control studies. The resulting cell–VitroGel mixture was mixed by pipetting and stabilized on ice for 15 min before being transferred to a syringe for animal injection.

### Micropillar‐Based Localized Stiffness Assay

Seventy thousand cancer cells were allowed to adhere on 2 µm or 4 µm polydimethyl siloxane (PDMS) pillars fabricated on coverslides (4D‐Cell, France) for 4–6 h; then, coverslides were fixed in 4% paraformaldehyde and immunostaining was performed. The stiffness of the PDMS micropillars is controlled by their size.

### EGFR Homodimerization Assay

Indicated cells were seeded on 0.5 kPa or 30 kPa coated wells for 24–36 h until cells reached 70–80% confluency. These cells were treated with EGF (5 ng/mL), sAgrin (1 µg/mL), or their combination for 30 min. Subsequently, the cells were washed with ice‐cold PBS and incubated with 1 ml/well of 2 mM cross‐linker BS3 for 30 min at room temperature. The cross‐linking reaction was terminated by adding 1 ml of 100 mM Tris (pH 7.5), followed by incubation at room temperature for 15 min. Whole‐cell lysates were prepared and analyzed by a Western blot on a 3–4%% SDS‐PAGE gel.

### Statistical Analysis—Preprocessing of Data

Preprocessing of the data was not performed. Outliers were not excluded from the analysis. For in vivo experiments, no animals were excluded from analysis and the sample size was determined using power analysis. The age and sex of animals are mentioned in the mice section. Randomization was not applied for experiments using cell lines. Mice were randomly assigned to treatment groups once they had 100–150 mm^3^ tumors.

#### Statistical Analysis—Data Presentation

Data are presented as the mean ± standard deviation (SD) or standard error of the mean (SEM) as indicated in figure legends.

#### Statistical Analysis—Sample Size Determination

The number of biological and technical repeats for each experiment is as indicated in the figure legends. For most in vitro experiments, three biological repeats were performed unless stated differently in the legend. Three biological replicates were chosen for in vitro experiments as this adheres to commonly held practice in biomedical research. Data are represented from one of three biological replicates unless stated otherwise in the respective figure legends. Normality of the data distribution was assessed using the Shapiro–Wilk test. For non‐normally distributed data, non‐parametric tests (Mann–Whitney U test or Kruskal–Wallis's test) were applied. A *p*‐value < 0.05 was considered statistically significant. All statistical tests were two‐sided and two‐tailed Students’ t‐test were used for most analysis. Outliers were not excluded in any analysis. The Exact *p*‐values are reported in the figures or figure legends, and *p* < 0.05, *p* < 0.01, and *p* < 0.001 are represented by *, **, and ***, respectively. Power analysis and sample size determination for in vivo studies: To estimate the minimum number of animals required for comparing either control or agrin‐depleted conditions, equal enrollment amongst groups and a 50% reduction in tumor size with error rate α = 0.05 and power of 80% were assumed. Using these parameters, a minimum sample size of *n* = 5 animals per group was obtained. To increase statistical rigor and confidence in the analysis, several animals per condition that equaled or exceeded the power analysis requirements for all the in vivo studies were employed.

#### Statistical Analysis—Statistical Methods Used

For comparisons between two groups, two‐tailed unpaired Student's t‐tests were used. For multiple group comparisons, one‐way ANOVA followed by Tukey's post‐hoc test or Dunnett's multiple comparison test was employed, as appropriate. Two‐way ANOVA was used for analyses involving two independent variables. Pearson's correlation coefficient was calculated to assess the relationship between continuous variables. Kaplan–Meier survival analysis with log‐rank test was used to evaluate overall survival and disease‐free survival.

#### Statistical Analysis—Software Used for Statistical Analysis

All statistical analyses were performed using GraphPad Prism 9.0 software (GraphPad Software, Inc., San Diego, CA, USA). For in vivo experiments, sample sizes were determined using G*Power 3.1 software, assuming a medium effect size (Cohen's d = 0.5), α = 0.05, and power (1‐β) = 0.8.

## Conflict of Interest

The authors declare no conflict of interest.

## Author Contributions

R.B.M., D.S., and P.E. contributed equally to this work and share first authorship. S.C. conceptualized the study. R.B.M., D.S., P.E., M.Y.O., D.B., G.T.B., A.S., C.C.Y., R.K., E.K., X.L., and S.C. conducted the formal analysis and investigation. D.B., P.E., R.B.M., and S.C. performed the in vivo experimentation. H.Y. and S.C. carried out the formal statistical analysis. G.T.B., B.F., F.L., J.Y.P., Z.W.O., D.G., C.F.B., and W.H. provided resources, including tumor specimens and mouse models. S.C. and W.H. supervised the study. S.C. drafted the original manuscript with inputs from R.B.M. and W.H. S.C. managed the project and secured funding.

## Supporting information



Supporting Information

Supplemental Table 1

## Data Availability

The data that support the findings of this study are available in the supplementary material of this article.
