# Supplementary material for: An Agrin–YAP/TAZ Rigidity Sensing Module Drives EGFR‐Addicted Lung Tumorigenesis
Source: Adv Sci (Weinh). 2025 Mar 31;12(20):2413443. doi: 10.1002/advs.202413443 (PMC12120797; doi:10.1002/advs.202413443)
Supplement: Supplementary file 1 — Supporting Information [file ADVS-12-2413443-s001.pdf]

ADVANCED SCIENCE
Open Access

Supporting Information

for *Adv. Sci.*, DOI 10.1002/advs.202413443

An Agrin–YAP/TAZ Rigidity Sensing Module Drives EGFR-Addicted Lung Tumorigenesis

Reza Bayat Mokhtari, Divyaleka Sampath, Paige Eversole, Melissa Ong Yu Lin, Dmitriy A. Bosykh, Gandhi T.K. Boopathy, Aravind Sivakumar, Cheng-Chun Wang, Ramesh Kumar, Joe Yeong Poh Sheng, Ellen Karasik, Barbara A. Foster, Han Yu, Xiang Ling, Wenjie Wu, Fengzhi Li, Zoë Weaver Ohler, Christine F. Brainson, David W. Goodrich, Wanjin Hong and Sayan Chakraborty**

Supporting Information:

AN AGRIN–YAP/TAZ RIGIDITY SENSING MODULE DRIVES EGFR-ADDICTED LUNG TUMORIGENESIS

Reza B. Mokhtari[1#], Divyaleka Sampath[2#], Paige Eversole[1#], Melissa Yu Lin Ong[2], Dmitriy A. Bosykh[1], Gandhi T.K. Boopathy[2], Aravind Sivakumar[2], Cheng-Chun Wang[2], Ramesh Kumar[2], Joe Yeong Poh Sheng[2], Ellen Karasik[1], Barbara A. Foster[1], Han Yu[3], Xiang Ling[1], Wenjie Wu[1], Fengzhi Li[1], Zoë Weaver Ohler[4], Christine F. Brainson[5], David Goodrich[1], Wanjin Hong[2*], and Sayan Chakraborty[1,6,7*]

#Equal contribution and co-first authors

***Correspondence:**

Wanjin Hong, Email: mcbhwj@imcb.a-star.edu.sg

Sayan Chakraborty, Email: Sayan.Chakraborty@RoswellPark.org

Figure S1

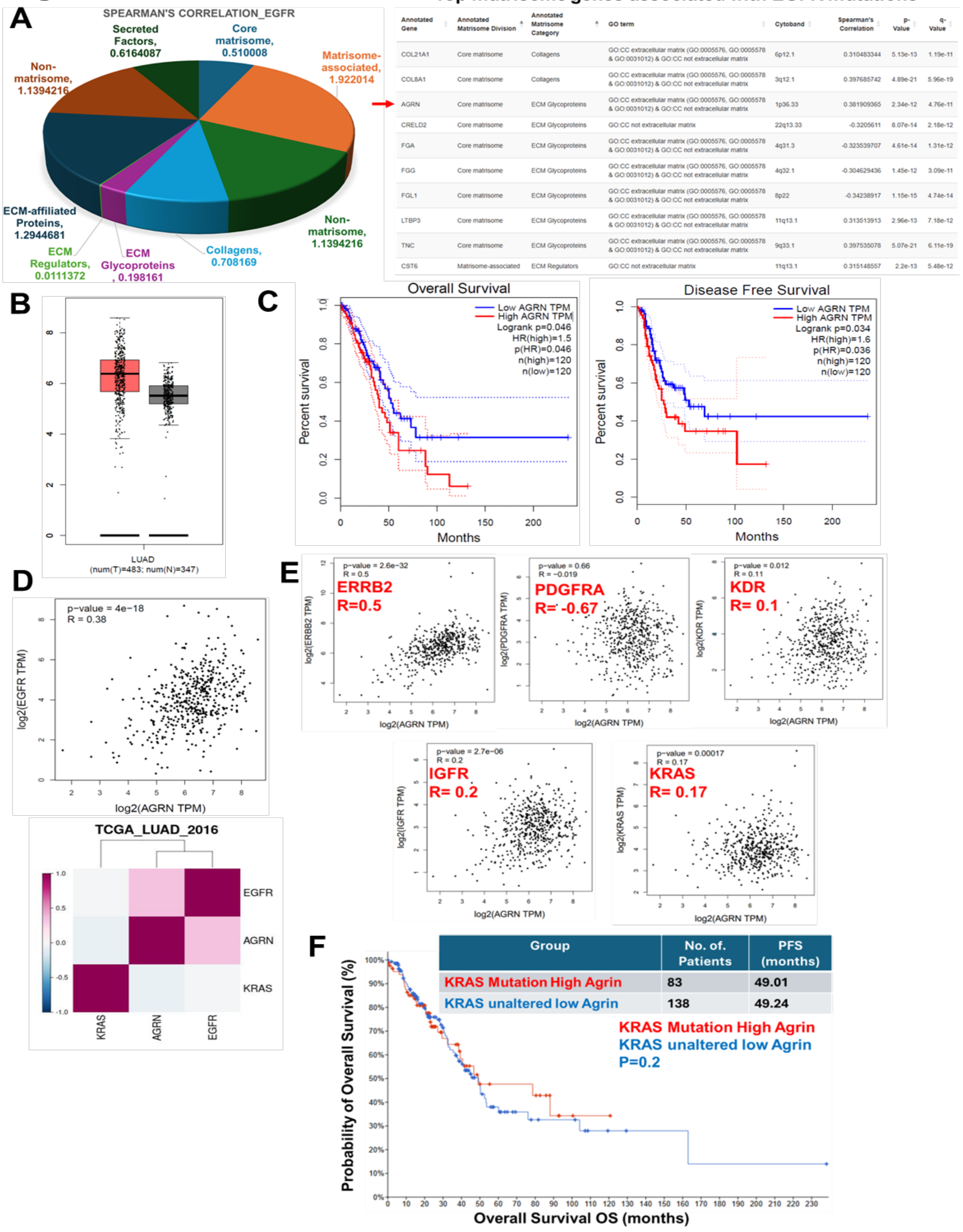

Annotated Gene	Annotated Matrisome Division	Annotated Matrisome Category	GO term	Cytoband	Spearman's Correlation	p-Value	q-Value
COL21A1	Core matrisome	Collagens	GO:CC extracellular matrix (GO:0005576, GO:0005578 & GO:0031012) & GO:CC not extracellular matrix	6p12.1	0.310483344	5.13e-13	1.19e-11
COL8A1	Core matrisome	Collagens	GO:CC extracellular matrix (GO:0005576, GO:0005578 & GO:0031012) & GO:CC not extracellular matrix	3q12.1	0.397685742	4.89e-21	5.96e-19
AGRN	Core matrisome	ECM Glycoproteins	GO:CC extracellular matrix (GO:0005576, GO:0005578 & GO:0031012) & GO:CC not extracellular matrix	1p36.33	0.381909365	2.34e-12	4.76e-11
CRELD2	Core matrisome	ECM Glycoproteins	GO:CC not extracellular matrix	22q13.33	-0.3205611	8.07e-14	2.18e-12
FGA	Core matrisome	ECM Glycoproteins	GO:CC extracellular matrix (GO:0005576, GO:0005578 & GO:0031012) & GO:CC not extracellular matrix	4q31.3	-0.323539707	4.61e-14	1.31e-12
FGG	Core matrisome	ECM Glycoproteins	GO:CC extracellular matrix (GO:0005576, GO:0005578 & GO:0031012) & GO:CC not extracellular matrix	4q32.1	-0.304629436	1.45e-12	3.09e-11
FGL1	Core matrisome	ECM Glycoproteins	GO:CC extracellular matrix (GO:0005576, GO:0005578 & GO:0031012) & GO:CC not extracellular matrix	8p22	-0.34238917	1.15e-15	4.74e-14
LTBP3	Core matrisome	ECM Glycoproteins	GO:CC extracellular matrix (GO:0005576, GO:0005578 & GO:0031012) & GO:CC not extracellular matrix	11q13.1	0.313513913	2.96e-13	7.18e-12
TNC	Core matrisome	ECM Glycoproteins	GO:CC extracellular matrix (GO:0005576, GO:0005578 & GO:0031012) & GO:CC not extracellular matrix	9q33.1	0.397535078	5.07e-21	6.11e-19
CST6	Matrisome-associated	ECM Regulators	GO:CC not extracellular matrix	11q13.1	0.315148557	2.2e-13	5.48e-12

Group	No. of. Patients	PFS (months)
KRAS Mutation High Agrin	83	49.01
KRAS unaltered low Agrin	138	49.24

Figure. S1. Clinical relevance of agrin–EGFR coexpression in LUAD. (A) Distribution of ECM-associated genes co-expressed with EGFR mutations in LUAD_TCGA datasets. Spearman's r values are shown for each subclass. The right panel shows the tabular presentation of top-matrisome associated genes significantly correlated with EGFR mutations. **(B)** Mean gene expression of AGRN in normal (N) (gray) versus lung adenocarcinoma (T) (red) tissues from the TCGA_Firehose legacy. **(C)** Overall survival (OS) and disease-free survival (DFS) based on high and low agrin gene expression amongst LUAD patients (GEPIA_database). Hazard ratios (HR) and P values are indicated for each panel. **(D)** Correlation analysis of epidermal growth factor receptor (EGFR) and agrin (AGRN) gene expressions in lung adenocarcinoma (LUAD) TCGA datasets (Spearman r = 0.38, *p* value indicated). A heatmap showing the degree of gene expression correlations between EGFR, AGRN, and KRAS in LUAD datasets. **(E)** Correlation analysis between agrin and receptor tyrosine kinases (RTKs) and KRAS in LUAD datasets (Spearman r and *p* values are shown for each gene). **(F)** OS curve for KRAS mutation patients stratified by high or low agrin expression. Progression free survival is also shown inset. (*p*-value indicated).

Figure S2

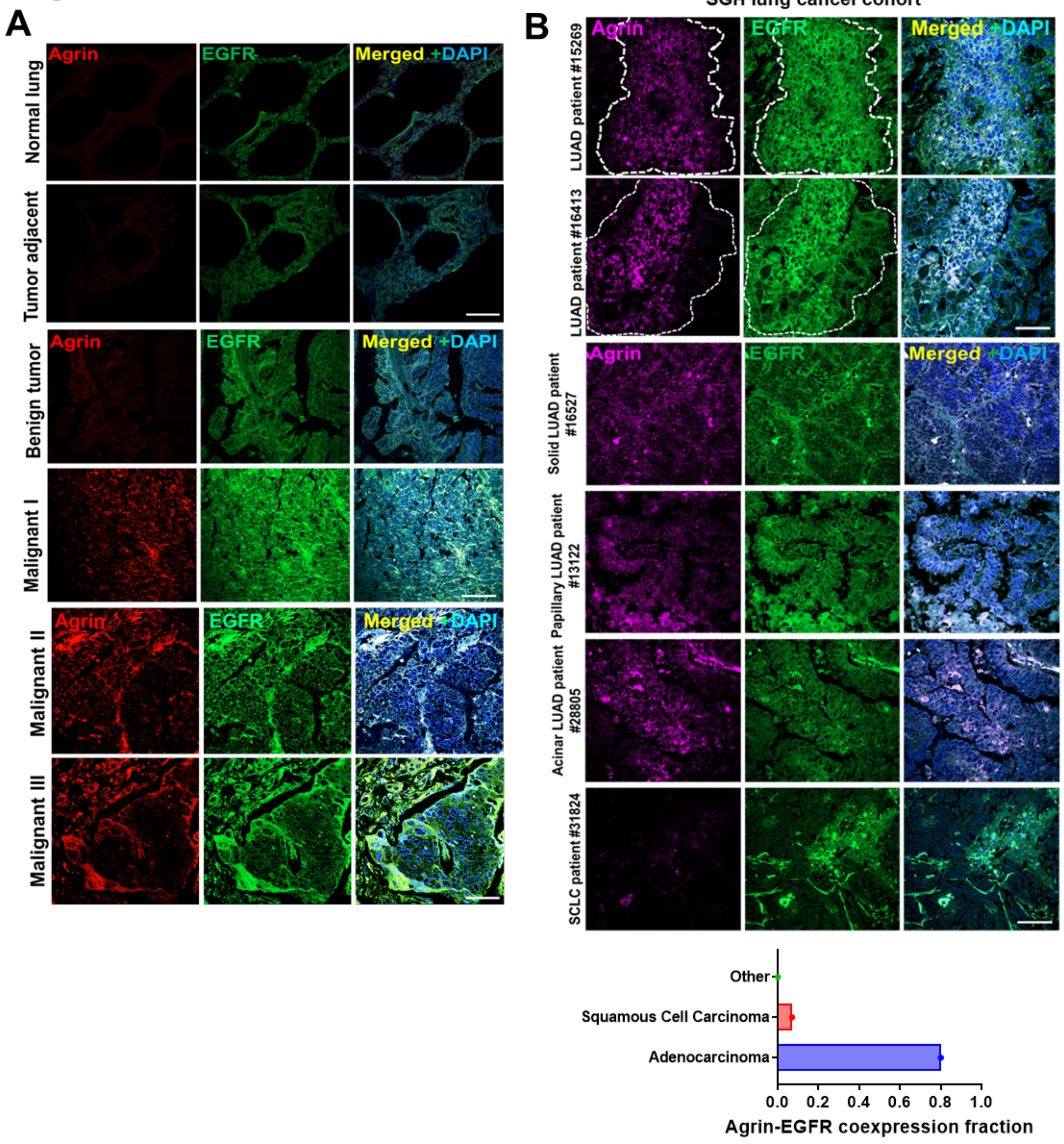

Figure. S2: Co-expression of agrin with EGFR in LUAD. (A–B) Representative confocal microscopy images of normal, tumor-adjacent, and malignant lung adenocarcinoma tissues showing agrin (red) and EGFR (green) expression, merged with DAPI (4′,6-diamidino-2-phenylindole) staining of the cancer cell nuclei (A). Panel B represents tissues from lung cancer patients from Singapore General Hospital (SGH). Scale bar: 100 μm (n = 7–10 tissues per condition) (B). Representative images of various subtypes of lung cancer tissues from Singapore General Hospital were stained similarly as in (B) (n = 53 tumors analyzed).

Figure S3

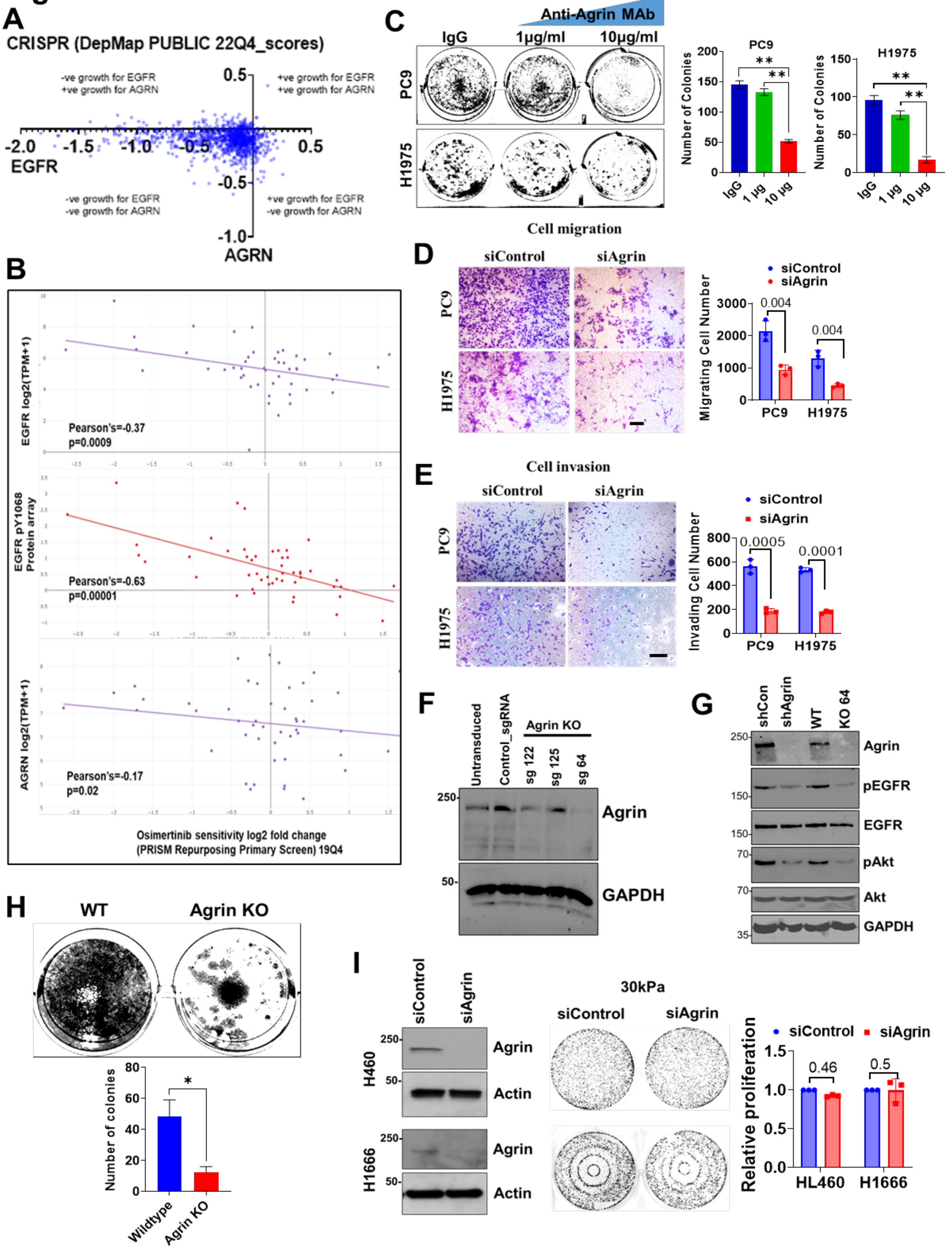
A
CRISPR (DepMap PUBLIC 22Q4_scores)
-ve growth for EGFR +ve growth for AGRN
+ve growth for EGFR +ve growth for AGRN
-ve growth for EGFR -ve growth for AGRN
+ve growth for EGFR -ve growth for AGRN
EGFR
AGRN
B
EGFR log2(TPM+1)
Pearson's=-0.37 p=0.0009
EGFR pY1068 Protein array
Pearson's=-0.63 p=0.00001
AGRN log2(TPM+1)
Pearson's=-0.17 p=0.02
Osimertinib sensitivity log2 fold change (PRISM Repurposing Primary Screen) 19Q4
C
Anti-Agrin MAb
IgG 1μg/ml 10μg/ml
PC9
H1975
Number of Colonies
IgG 1 μg 10 μg
Cell migration
D
siControl siAgrin
Migrating Cell Number
0.004
0.004
Cell invasion
E
Invading Cell Number
0.0005
0.0001
F
Untransduced
Control_sgRNA
Agrin KO
sg 122 sg 125 sg 64
Agrin
GAPDH
G
shCon shAgrin WT KO 64
Agrin
pEGFR
EGFR
pAkt
Akt
GAPDH
H
WT Agrin KO
Number of colonies
Wildtype Agrin KO
I
siControl siAgrin
H460
H1666
Agrin
Actin
30kPa
Relative proliferation
0.46
0.5
HL460 H1666

Figure. S3. Blocking agrin inhibits lung cancer cell proliferation, migration, and invasion. (A) DepMap analysis showing the distribution of agrin as a co-dependent gene in EGFR-depleted cell lines. **(B)** DepMap plot showing the expression of phosphorylated and total EGFR and agrin in response to osimertinib (respective *p* values and Pearson's r indicated). **(C)** Indicated cell lines were cultured on 30 kPa substrates in the presence of increasing concentrations of anti-agrin antibody for 7 days. Representative bright-field microscopy images for colonies are shown, and mean colony numbers +/– SD are presented (n = 3, Student's t test, $^{**}p = 0.002$). **(D-E)** Representative crystal violet stained bright-field microscopy images of Boyden chamber migration and invasion assays in control and agrin-depleted cells. Scale bar: 50 μm. The number of migrating and invading cells are presented as the mean +/– SD (n = 3, Student's t test, *p* values indicated). **(F)** Western blot comparing the shControl, shAgrin versus wild type (WT), and agrin KO#64 PC9 cells for the indicated EGFR downstream signaling proteins (n = 2 repeats). **(G)** Western blot comparisons of agrin-silenced versus knockout PC9 cells for the indicated EGFR signaling proteins. GAPDH served as a loading control. **(H)** Colony formation assay of WT and agrin KO PC9 cells on 30 kPa substrates imaged after 7 days. The number of colonies presented as the mean+/– SD (n = 3, Student's t test, $^{*}p = 0.01$). **(I)** Western blot showing agrin knockdown (left panels) and colony formation assay for control and agrin silenced HL460 and H1666 cell lines (low EGFR status) on 30 kPa substrates imaged after 7 days. The number of colonies presented as the mean+/– SD (n = 3, Student's t test, *p* value = 0.5; 0.46, respectively).

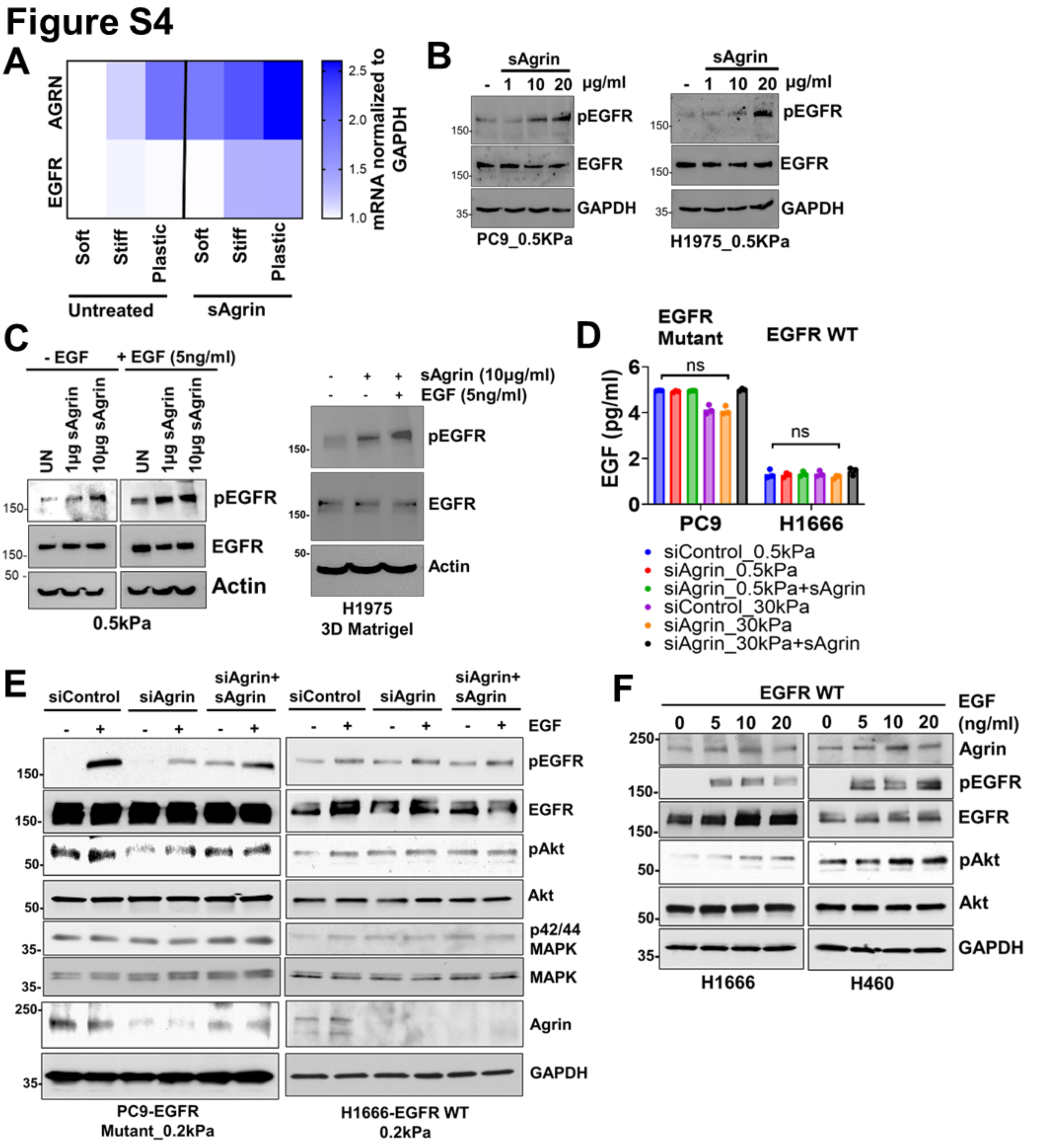

Figure. S4. Regulation of agrin on EGF-induced EGFR activation in EGFR mutant and WT cell lines. (**A**) Heatmap showing AGRN and EGFR mRNA levels in PC9 cells seeded on different substrate with or without 10 µg/mL sAgrin for 18 h (n = 3 replicates). (**B**) Indicated cell lines seeded on 0.5 kPa substrates were treated with

increasing concentrations of sAgrin for 18 h. The cell lysates were analyzed by Western blotting for EGFR phosphorylation. GAPDH served as a loading control (experiment repeated three times). (**C**) PC9 cells cultured on 0.5 kPa with increasing concentrations of sAgrin for 18 h were treated with EGF or no EGF for 1 h. For the right panel, H1975 cells were grown in Matrigel alone or in combination with sAgrin and EGF for 2 days. The whole cell lysates were analyzed as in (B). β-Actin served as a loading control (experiment repeated three times). **(D)** EGF-ELISA analysis of agrin-silenced cells cultured on soft or stiff matrix with or without 10μg/ml sAgrin for 16h (n=3, two-way ANOVA, ns). **(E)** Agrin knockdown PC9 and H1666 cells cultured on 0.2kPa substrates were treated with 5 ng/ml EGF for 30 min alone or with prior sAgrin treatment (for 16h). Cell lysates were analyzed by Western blot for the indicated proteins. GAPDH served as a loading control (experiment repeated three times). **(F)** The indicated EGFR-WT cell lines were treated with increasing concentrations of EGF for 60 min. Resulting cell lysates were analyzed by Western blot for the indicated proteins. GAPDH served as a loading control (experiment repeated three times).

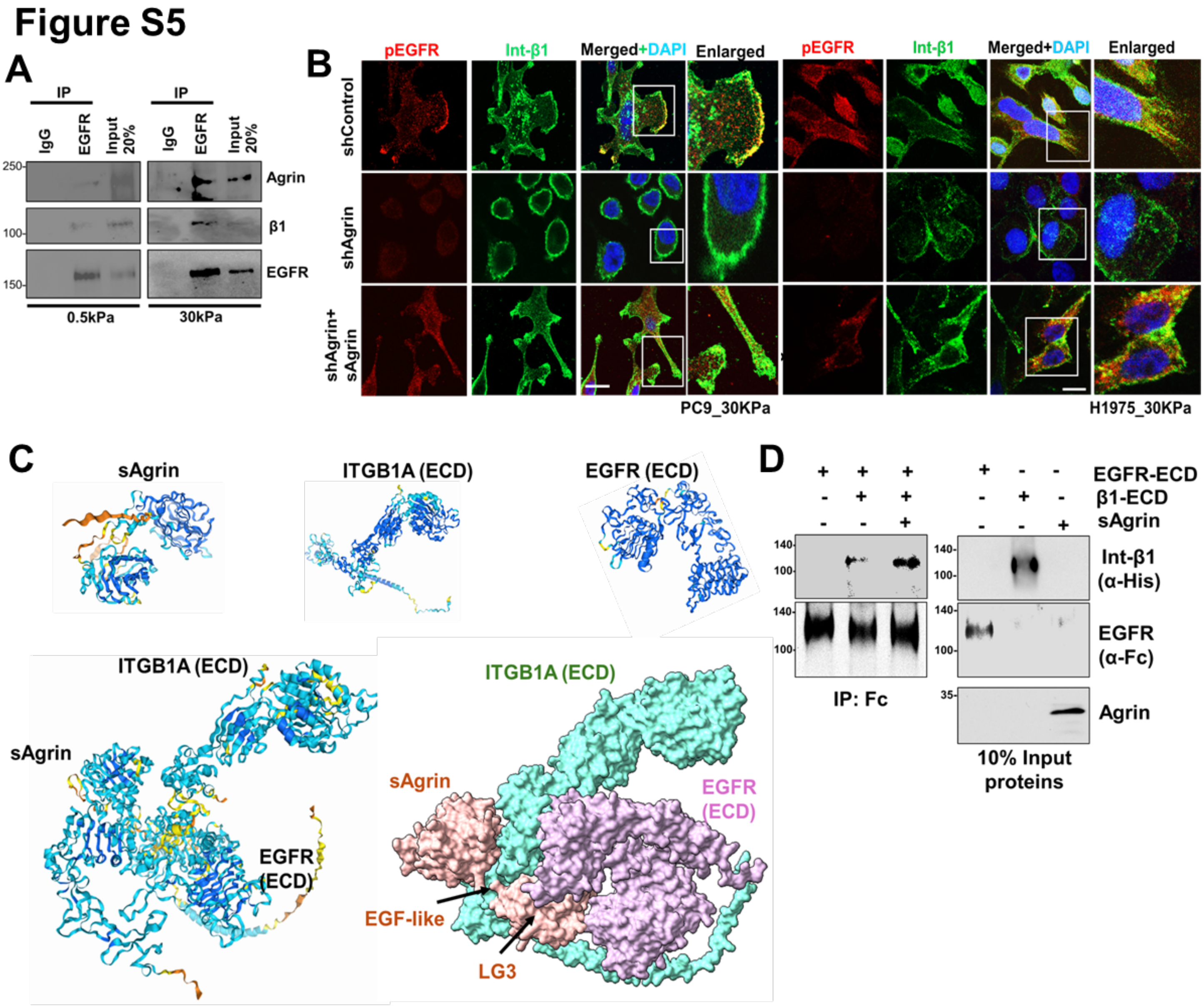

Figure. S5: Agrin bridges EGFR-integrin to form a mechanosensitive complex. (A) H1975 cell lysates on 0.5 kPa or 30 kPa were immunoprecipitated with an isotype control (IgG) or EGFR antibody and Western blotted for agrin and integrin β1. The blots were stripped and probed for EGFR. As input controls, 20% whole cell lysates were run (experiment repeated two times). **(B)** Representative confocal images of control and agrin knockdown cells and those treated with 10 μg/ml sAgrin for 18 h on 30 kPa substrates showing pEGFR (red) and integrin β1 (green) immunostaining. Scale bar: 10 μm. Boxed region is presented as enlarged panels (experiment repeated

three times). **(C)** Molecular modeling of sAgrin and EGFR-Integrin β1 ECDs on ChimeraX. A side view of protein complexes is shown as protein chains (bottom left) or as trimeric polymer surface version (bottom right). **(D)** Two hundred micrograms of EGFR-Fc was immobilized to agarose A beads. The washed beads were treated with integrin β1-His (100 μg) in the presence or absence of sAgrin (100 μg) for 2h. Eluted protein complex was Western blotted for His and Fc, respectively (n=3 repeats). Twenty micrograms of each recombinant protein was run as input controls.

Figure S6

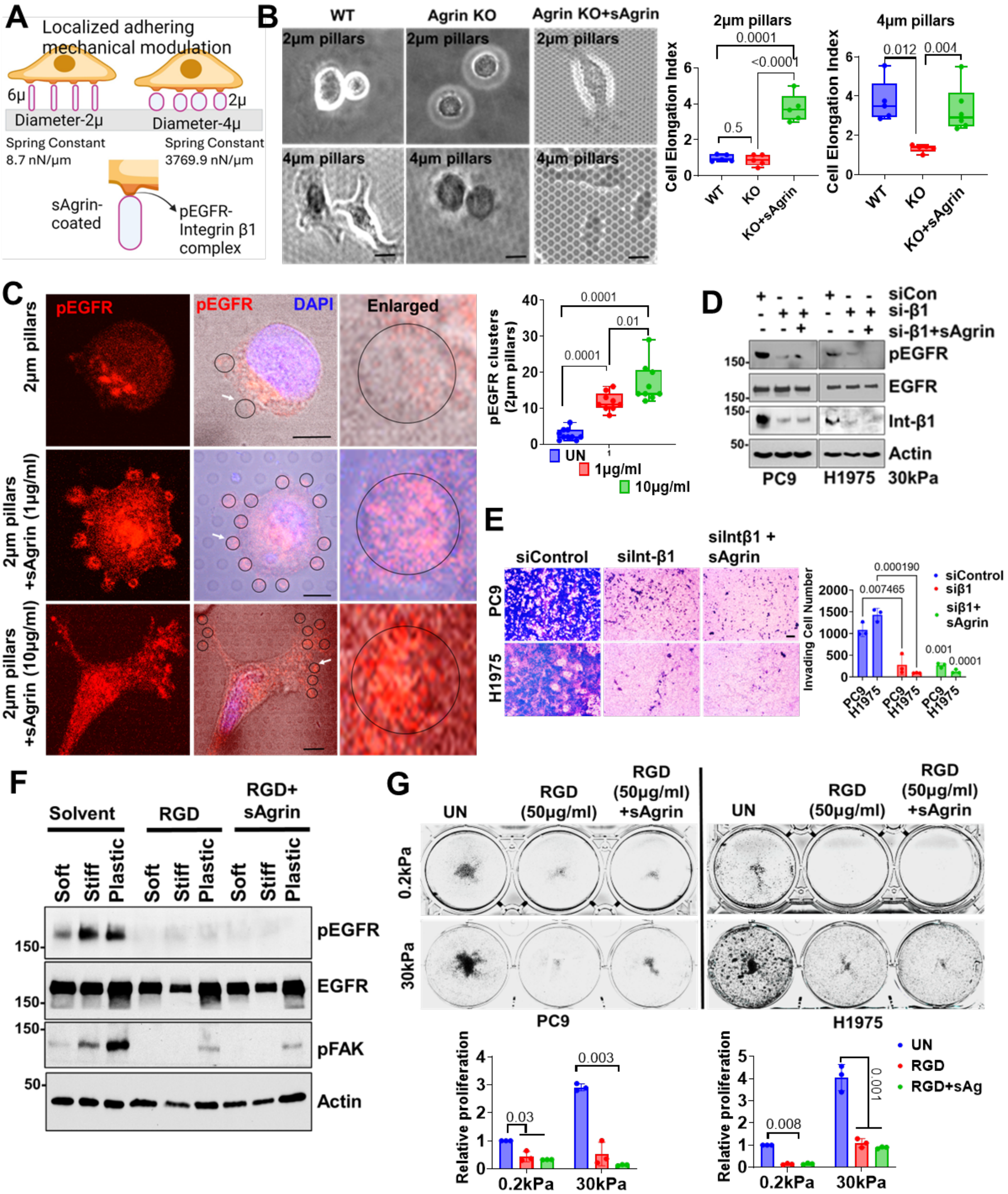

A
Localized adhering mechanical modulation
6μ
2μ
Diameter-2μ
Diameter-4μ
Spring Constant 8.7 nN/μm
Spring Constant 3769.9 nN/μm
sAgrin-coated
pEGFR-Integrin β1 complex
B
WT
Agrin KO
Agrin KO+sAgrin
2μm pillars
4μm pillars
Cell Elongation Index
0.0001
<0.0001
0.5
0.012
0.004
WT
KO
KO+sAgrin
C
pEGFR
DAPI
Enlarged
2μm pillars
2μm pillars +sAgrin (1μg/ml)
2μm pillars +sAgrin (10μg/ml)
pEGFR clusters (2μm pillars)
0.0001
0.01
UN
1μg/ml
10μg/ml
D
siCon
si-β1
si-β1+sAgrin
pEGFR
EGFR
Int-β1
Actin
PC9
H1975
30kPa
E
siControl
siInt-β1
siIntβ1 + sAgrin
PC9
H1975
Invading Cell Number
0.000190
0.007465
0.001
0.0001
siControl
siβ1
siβ1+ sAgrin
F
Solvent
RGD
RGD+ sAgrin
Soft
Stiff
Plastic
pEGFR
EGFR
pFAK
Actin
G
UN
RGD (50μg/ml)
RGD (50μg/ml) +sAgrin
0.2kPa
30kPa
PC9
H1975
Relative proliferation
0.03
0.003
0.008
0.001
RGD
RGD+sAg

Figure. S6: Integrin-β1 is essential for linking agrin-induced localized rigidity responses to EGFR-oncogenic action. (A-B) Schema showing the cell–micropillar interactions and spring's constant for micropillars. WT cells and agrin KO cells were seeded on 2 μm or 4 μm pillars either alone or containing 10 μg/mL sAgrin for 4 h. Representative bright-field images shown for each cell condition. Scale bar: 10 μm. The mean cell elongation index (ratio of length to width) +/-SD is shown for all conditions (n=6-10 cells per condition, Students t test, *p* values indicated). (**C**) PC9 cells were seeded on 2 μm (soft) pillars containing the indicated amounts of sAgrin for 4 h. Representative confocal images showing pEGFR status costained with DAPI. Black circle in the enlarged panels shows increased pEGFR staining intensities. The pEGFR clusters within each pillar (small black circles) are presented as the mean +/– SD (n = 10–15 cells, Student's t test, *p* values indicated). **(D)** Western blot analysis in indicated integrin β1-silenced cells alone or those treated with 10μg/ml sAgrin for 16h. Actin served as a loading control (n=2 repeats). **(E)** Cells from panel (D) were subjected to transwell invasion assays. Mean number of invading cells+/-SD were quantified (n=3, Students t test, *p* values shown). **(F)** Western blot in H1975 cells showing EGFR activation in control cells or those treated with RGD (50μM for 4h) cells alone or treated with 10μg/ml sAgrin on soft, stiff and plastic plates. pFAK activity was assessed to determine the integrin-blocking action by RGD peptides. Actin served as a loading control (n=3 repeats). **(G)** Representative images of colony formation in indicated cells on soft and stiff substrates from panel (F) treated with RGD for 5 days alone or in presence of sAgrin (n=3, two-way ANOVA, *p* values indicated).

Figure S7

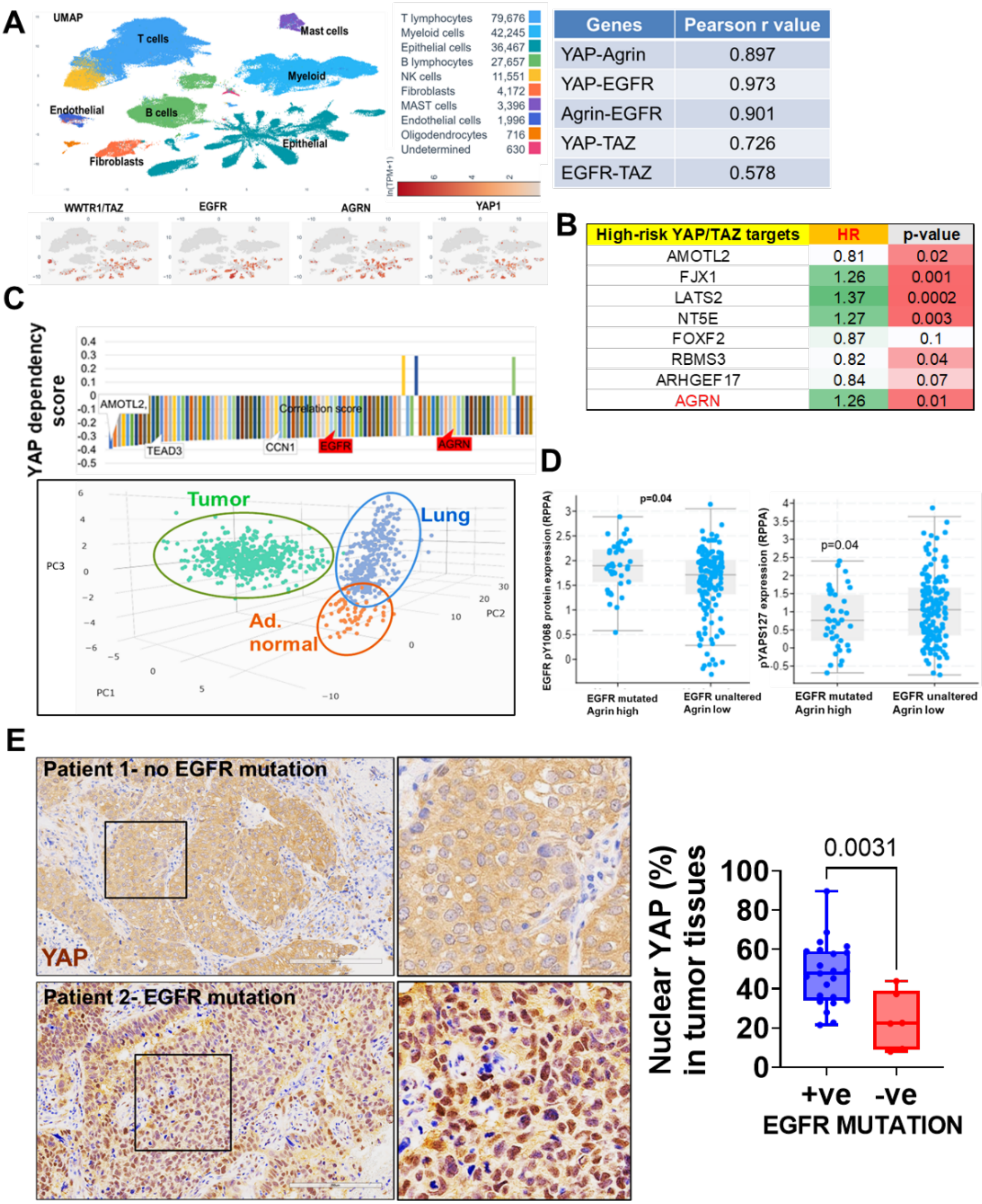
A
UMAP
T cells
Mast cells
Myeloid
Endothelial
B cells
Epithelial
Fibroblasts
T lymphocytes 79,676
Myeloid cells 42,245
Epithelial cells 36,467
B lymphocytes 27,657
NK cells 11,551
Fibroblasts 4,172
MAST cells 3,396
Endothelial cells 1,996
Oligodendrocytes 716
Undetermined 630
ln(TPM+1)
Genes | Pearson r value
YAP-Agrin 0.897
YAP-EGFR 0.973
Agrin-EGFR 0.901
YAP-TAZ 0.726
EGFR-TAZ 0.578
WWTR1/TAZ
EGFR
AGRN
YAP1
B
High-risk YAP/TAZ targets | HR | p-value
AMOTL2 0.81 0.02
FJX1 1.26 0.001
LATS2 1.37 0.0002
NT5E 1.27 0.003
FOXF2 0.87 0.1
RBMS3 0.82 0.04
ARHGEF17 0.84 0.07
AGRN 1.26 0.01
C
YAP dependency score
AMOTL2
TEAD3
CCN1
Correlation score
EGFR
AGRN
Tumor
Lung
Ad. normal
PC1
PC2
PC3
D
EGFR pY1068 protein expression (RPPA)
p=0.04
pYAPS127 expression (RPPA)
EGFR mutated Agrin high
EGFR unaltered Agrin low
E
Patient 1- no EGFR mutation
YAP
Patient 2- EGFR mutation
Nuclear YAP (%) in tumor tissues
0.0031
+ve
-ve
EGFR MUTATION

Figure. S7. Relevance of agrin–EGFR–YAP/TAZ in LUAD patient specimens. (A) U-MAP plot showing the co-distribution of TAZ, EGFR, agrin, and YAP1 in the epithelial cells of the lung adenocarcinoma sc-RNA sequencing dataset. **(B)** Development of a high-risk YAP/TAZ target gene signature predicting survival outcomes in LUAD patients. **(C)** DepMap scores of YAP/TAZ target genes for YAP dependency (upper panels). EGFR and AGRN are highlighted in red. Principal component analysis (PCA) of YAP/TAZ target gene expression in normal lung, tumor-adjacent, and lung adenocarcinoma (TCGA_LUAD_GEPIA). **(D)** Status of pEGFR and pYAP in LUAD_TCGA patients datasets stratified based on high agrin–EGFR mutation and low agrin and unaltered EGFR (n = 230, cBioportal, Mann–Whitney Test, *p* values indicated). **(E)** Representative IHC images of nuclear YAP in LUAD patients from Roswell Park repository. The nuclear YAP staining intensities are represented as the mean+/-SD (n=20 patients, Students t test, *p* value presented).

Figure S8

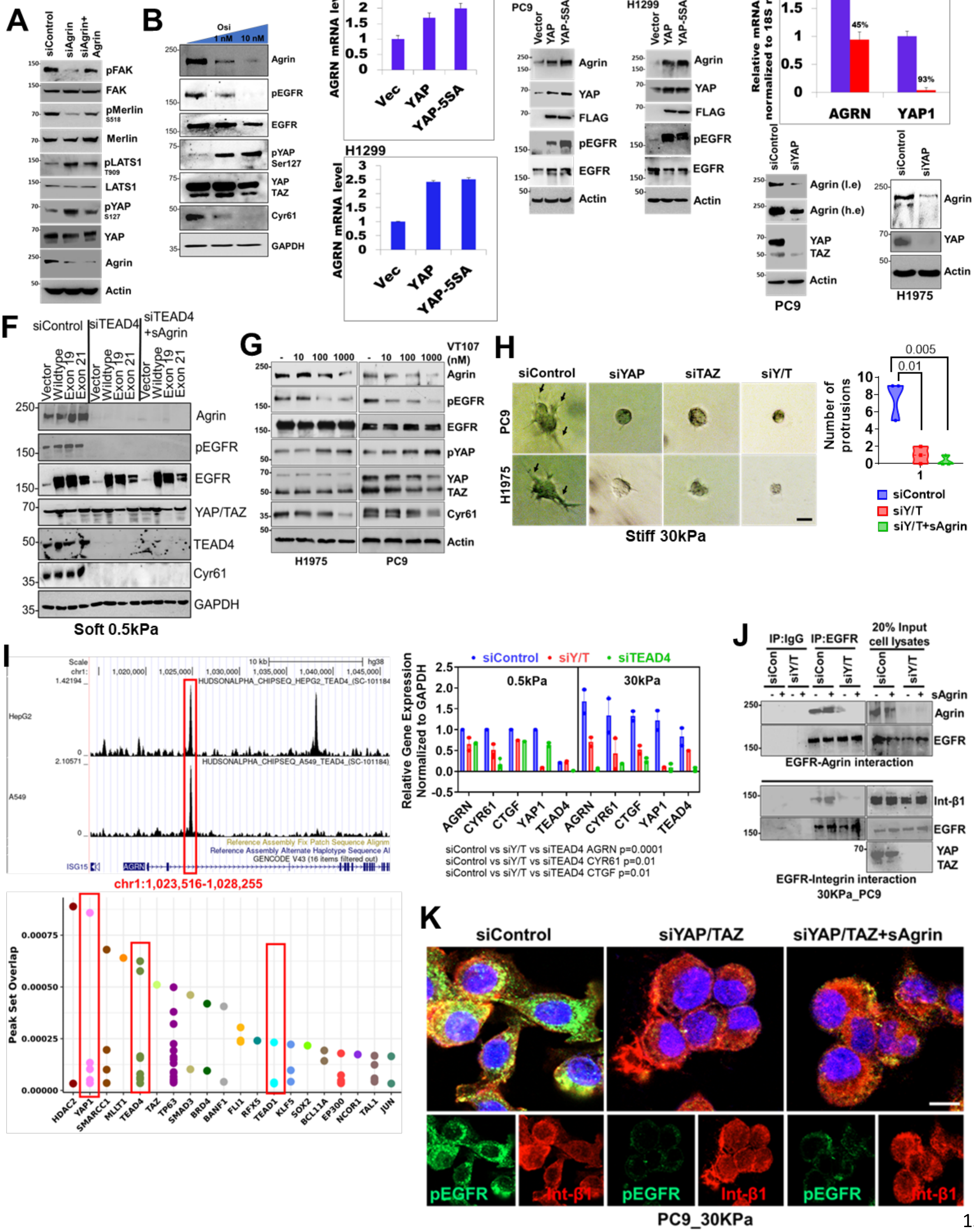
A
siControl
siAgrin
siAgrin+ Agrin
pFAK
FAK
pMerlin S518
Merlin
pLATS1 T909
LATS1
pYAP S127
YAP
Agrin
Actin
B
Osi
1 nM
10 nM
Agrin
pEGFR
EGFR
pYAP Ser127
YAP
TAZ
Cyr61
GAPDH
C
PC9
AGRN mRNA level
Vec
YAP
YAP-5SA
H1299
D
PC9
H1299
Vector
YAP
YAP-5SA
Agrin
YAP
FLAG
pEGFR
EGFR
Actin
E
Relative mRNA normalized to 18S rRNA
siControl
siYAP
45%
93%
AGRN
YAP1
siControl
siYAP
Agrin (l.e)
Agrin (h.e)
YAP
TAZ
Actin
PC9
Agrin
YAP
Actin
H1975
F
siControl
siTEAD4
siTEAD4 +sAgrin
Vector
Wildtype
Exon 19
Exon 21
Agrin
pEGFR
EGFR
YAP/TAZ
TEAD4
Cyr61
GAPDH
Soft 0.5kPa
G
VT107 (nM)
10
100
1000
Agrin
pEGFR
EGFR
pYAP
YAP
TAZ
Cyr61
Actin
H1975
PC9
H
siControl
siYAP
siTAZ
siY/T
PC9
H1975
Stiff 30kPa
Number of protrusions
0.005
0.01
siControl
siY/T
siY/T+sAgrin
I
HUDSONALPHA_CHIPSEQ_HEPG2_TEAD4_(SC-101184
HUDSONALPHA_CHIPSEQ_A549_TEAD4_(SC-101184)
HepG2
A549
Reference Assembly Fix Patch Sequence Alignm
Reference Assembly Alternate Haplotype Sequence Al
GENCODE V43 (16 items filtered out)
ISG15
AGRN
chr1:1,023,516-1,028,255
siControl
siY/T
siTEAD4
0.5kPa
30kPa
Relative Gene Expression Normalized to GAPDH
AGRN
CYR61
CTGF
YAP1
TEAD4
siControl vs siY/T vs siTEAD4 AGRN p=0.0001
siControl vs siY/T vs siTEAD4 CYR61 p=0.01
siControl vs siY/T vs siTEAD4 CTGF p=0.01
Peak Set Overlap
HDAC2
YAP1
SMARCC1
MLLT1
TEAD4
TAZ
TP63
SMAD3
BRD4
BANF1
FLI1
RFX5
TEAD1
KLF5
SOX2
BCL11A
EP300
NCOR1
TAL1
JUN
J
IP:IgG
IP:EGFR
20% Input cell lysates
siCon
siY/T
sAgrin
Agrin
EGFR
EGFR-Agrin interaction
Int-β1
EGFR
YAP
TAZ
EGFR-Integrin interaction 30KPa_PC9
K
siControl
siYAP/TAZ
siYAP/TAZ+sAgrin
pEGFR
Int-β1
PC9_30KPa

Figure. S8. YAP/TAZ control agrin and its mechanical rigidity effects on EGFR–integrin activation. (**A**) Western blot results in the control and agrin depleted, and those treated with sAgrin for the indicated Hippo pathway proteins. β-Actin served as a loading control (experiment repeated three times). (**B**) PC9 cells were treated with increasing dose of osimertinib for 18 h before analyzing the cell lysates for the indicated proteins by Western blot. GAPDH served as a loading control (experiment repeated twice). (**C**) AGRN mRNA levels determined by RT-PCR analysis in the indicated cell lines expressing a vector control, YAP1, or YAP5SA lentiviral constructs (experiment performed in triplicate). (**D**) Cell lysates from (C) were immunoblotted for the indicated proteins. β-Actin served as a loading control (experiment repeated three times). (**E**) RT-PCR analysis on control and YAP1 depleted PC9 cells showing AGRN and YAP1 expressions normalized to 18s rRNA (experiment performed in triplicates). Western blot analysis in the indicated cell lysates from control and siYAP conditions for the indicated proteins. β-Actin served as a loading control (experiment repeated three times). (**F**) H1299 expressing vector, EGFR, or its mutants DEL19 or 21 were treated with control or TEAD4 siRNA. One batch of TEAD4 knockdown cells were treated with sAgrin (10 µg/mL) for 18 h. Cell lysates were analyzed by Western blotting for the indicated proteins. GAPDH served as a loading control (experiment repeated twice). (**G**) Indicated cell lines were treated with increasing concentrations of VT107 for 18 h. Subsequently, whole cell lysates were analyzed by Western blotting for the indicated proteins. β-Actin served as a loading control (experiment repeated three times). **(H)** Indicated cell lines transfected with control, siYAP, siTAZ, and siYAP/TAZ were seeded on 30 kPa stiff collagen matrix for 48 h. Representative bright-field microscopy images show spheroids with invasive protrusions (marked by black arrows). The number of protrusions is presented as the mean +/– SD (n = 3, unpaired Student's t test, *p* values indicated). **(I)** Visualization of TEAD4 enrichments in various cell lines on second intron of AGRN gene using UCSC genome browser CistromeDB (left top panel). The enlarged gene region (red) was analyzed using http://dbtoolkit.cistrome.org/ for peak distribution of putative transcription factor(s) binding (left bottom panel). (Right panel) PC9 cells on indicated substrates were treated with control, YAP/TAZ and TEAD4 siRNAs. Three days later, total mRNA was subjected to RT-PCR analysis for the indicated Y/T target genes (n=3, one way ANOVA, p values indicated). (**J**) Control and Y/T-depleted PC9

cells on 30 kPa were immunoprecipitated with EGFR antibody. Resulting lysates were probed for agrin, integrin β1, and EGFR. Twenty percent whole cell lysates served as input. The YAP/TAZ panel verifies the knockdown in lysates used for Co-IP. **(K)** Control, YAP/TAZ depleted and those treated with 10 μg/ml sAgrin for 18h were cultured on 30kPa. Representative confocal images showing pEGFR (green) and integrin β1 (red) are presented. Scale bar: 10μm (experiment repeated three times).

Figure S9

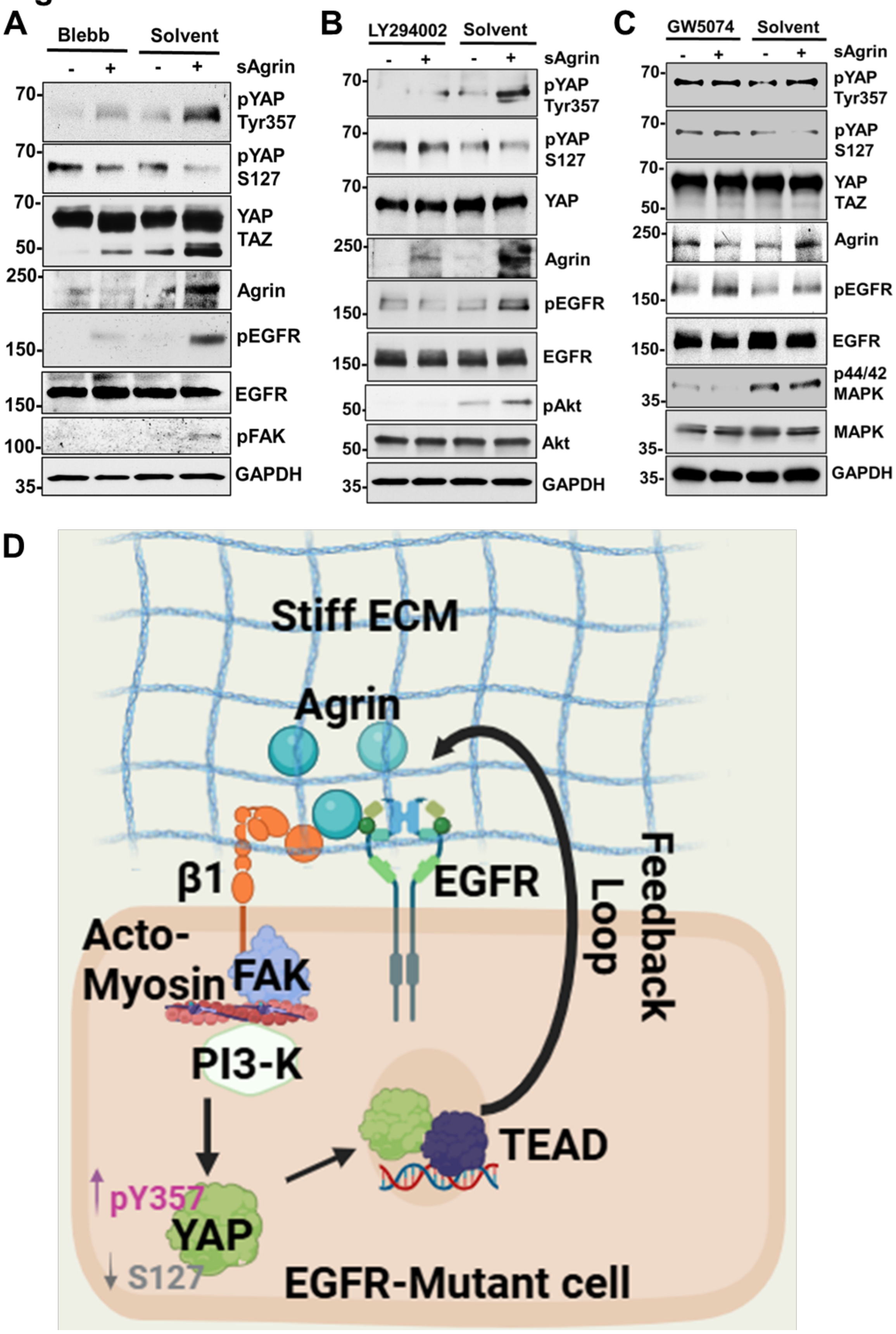
A
Blebb
Solvent
sAgrin
pYAP Tyr357
pYAP S127
YAP
TAZ
Agrin
pEGFR
EGFR
pFAK
GAPDH
B
LY294002
Solvent
sAgrin
pYAP Tyr357
pYAP S127
YAP
Agrin
pEGFR
EGFR
pAkt
Akt
GAPDH
C
GW5074
Solvent
sAgrin
pYAP Tyr357
pYAP S127
YAP
TAZ
Agrin
pEGFR
EGFR
p44/42 MAPK
MAPK
GAPDH
D
Stiff ECM
Agrin
β1
EGFR
Feedback Loop
Acto-Myosin
FAK
PI3-K
TEAD
pY357
YAP
S127
EGFR-Mutant cell

Figure. S9. Impact of actin-myosin, PI3-K and RAF-MAPK inhibition on YAP/TAZ feedback on agrin-EGFR. (A-C) Western blot analysis for the indicated proteins in H1975 cell lysates cultured on 0.2kPa substrates were treated with blebbistatin, LY294002, and GW-5074 (1μM, 6h) alone or pre-treated with sAgrin overnight. GAPDH served as loading controls. Each experiment was repeated at least two times. **(D)** Working model of FAK-PI3-K-actomyosin dependent activation of YAP/TAZ by agrin-EGFR mechanosensitive complex.

Figure S10

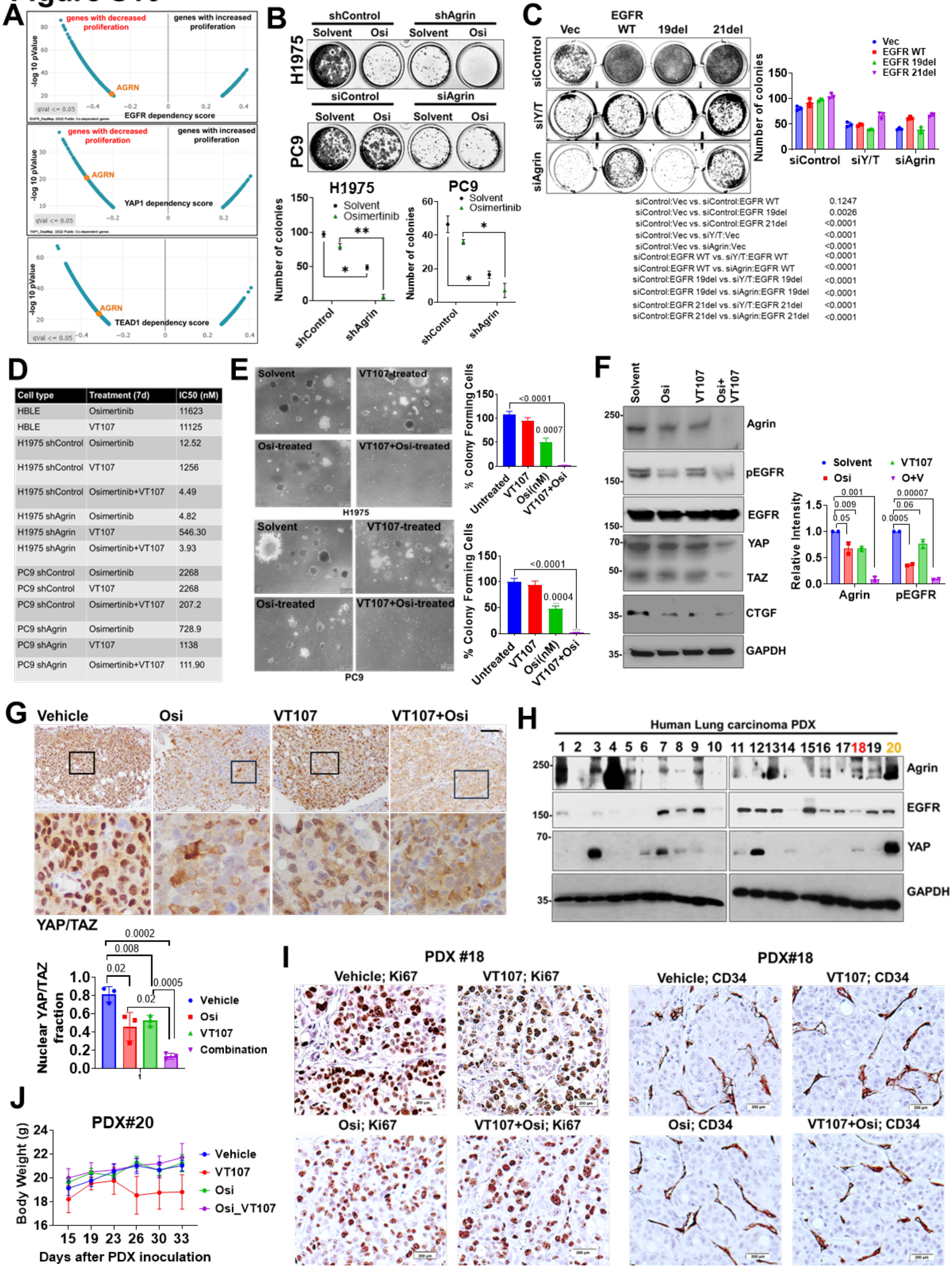

Cell type	Treatment (7d)	IC50 (nM)
HBLE	Osimertinib	11623
HBLE	VT107	11125
H1975 shControl	Osimertinib	12.52
H1975 shControl	VT107	1256
H1975 shControl	Osimertinib+VT107	4.49
H1975 shAgrin	Osimertinib	4.82
H1975 shAgrin	VT107	546.30
H1975 shAgrin	Osimertinib+VT107	3.93
PC9 shControl	Osimertinib	2268
PC9 shControl	VT107	2268
PC9 shControl	Osimertinib+VT107	207.2
PC9 shAgrin	Osimertinib	728.9
PC9 shAgrin	VT107	1138
PC9 shAgrin	Osimertinib+VT107	111.90

Figure. S10. Combined EGFR–YAP/TEAD inhibition reduces agrin-dependent proliferation in vitro and in vivo. (**A**) DepMap analysis showing AGRN as a top co-dependent gene for EGFR, YAP1, and TEAD1 (q values indicated). (**B**) Colony formation assay images in control and agrin depleted cells treated with solvent and osimeritinib (1 μM). The number of colonies is presented as the mean +/– SD (n = 3, ***p* = 0.002, **p* = 0.04, Student's t test). (**C**) Colony formation assay images of H1299-vector, EGFR-WT, 19del, and 21-point mutation silenced with a control, YAP/TAZ, and agrin siRNA pool. The number of colonies is presented as the mean +/– SD (n = 3, Tukey's multiple comparison test, *p* values indicated). (**D**) Determination of the IC50 in the indicated control and agrin depleted cell lines upon treatment with osimertinib and VT107. (**E**) Three-dimensional colony formation assay of indicated cell lines treated with solvent and respective IC50 concentrations of VT107, osimertinib, and their combination for 7 days. The number of active spheroids +/– SD is presented (n = 3 images per group, unpaired Student's t test, *p* values indicated). (**F**) PC9 cells treated with solvent, osimertinib, VT107, and their combinations for 1 day. Whole cell lysates were probed for the indicated proteins. GAPDH served as a loading control. Mean Agrin and pEGFR protein intensities+/-SD are shown (n=2, multiple t tests, *p* values indicated). (**G**) Representative IHC images of YAP/TAZ localization in the lung orthotopic tumors receiving VT107 and osimertinib and their combination therapies. The mean nuclear intensity +/– SD is shown (n = 3 tumors/group, multiple t tests, *p* values indicated). Scale bar: 100 μm. The boxed region is presented as enlarged panels. (**H**) Western blot analysis in human lung cancer PDXs for the indicated protein expression (experiment repeated two times). GAPDH served as loading control. (**I**) Representative IHC images of KI67 and CD-31 from mouse bearing PDX#18 tumors that received the same treatment as in Fig. 7E (n = 3 tumors from three mice/group). Scale bar: 200 μm. (**J**) Body weight of mice harboring PDX tumors presented as the mean +/– SEM on indicated days (n = 5 mice per group).

Table S1: List of genes correlated with high EGFR expression in LUAD (Excel sheet)

Table S2: List of shRNA/ sgRNA sequences used for lentiviral transduction

AGRN-1 shRNA	TGCTGTTGACAGTGAGCGAGCCAATACTGTGACTTCCAAATAGTGAAGCCACAGATG TATTTGGAAGTCACAGTATTGGCGTGCCTACTGCCTCGGA’
AGRN-2 shRNA	TGCTGTTGACAGTGAGCGAGCCAGTACACGCCTTTAAATATAGTGAAGCCACAGATG TATATTTAAAGGCGTGTACTGGCGTGCCTACTGCCTCGGA
Agrin sg_RNA	TGCTGCACCGGGTCCACGTT (F)<br>AACGTGGACCCGGTGCAGCA (R)

Table S3: List of RT-PCR primers used

AGRIN	5’ – ACA CCG TCC TCA ACC TGA AG – 3’ (F)<br>5’ – CCA GGT TGT AGC TCA GTT GC – 3’ (R)
CTGF	5'- GCC AAT GAG CTG AAT GGA GT - 3' (F)<br>5'- CAA TCC GGT GTG AGT TGA TG - 3' (R)
GAPDH	5’ - CTATAAATTGAGCCCGCAGC – 3’ (F)<br>5’ - GACCAAATCCGTTGACTCCG – 3’ (R)
EGFR	5’ – AACACCCTGGTCTGGAAGTACG – 3’ (F)<br>5’ – TCGTTGGACAGCCTTCAAGACC – 3’ (R)
	**TAQMAN probes**
YAP	Hs00371735_m1 YAP1 1071006 C6
TEAD	Hs00173359_m1 TEAD1, 532405A7
TAZ	Hs00794094_m1 TAZ 1118458 F12
CYR61	Hs00155479_m1 CYR61 FAM PN4351370
CTGF	Hs00170014_m1 CTGF FAM PN4351370
AGRIN	Hs00394748_m1 AGRN FAM PN4351370
18S	Hs03003631_g1 18S FAM PN4351370

Supplementary Experimental Procedures

Antibodies, reagents, and small molecule inhibitors

The following antibodies were used in the study: Mouse monoclonal anti-Agrin (D-2), Santa Cruz Biotechnology, Cat#sc-374117; RRID: AB_10947251 (WB dilution: 1:250, IF-IHC: I:50); Human Agrin clone PIF12 (in house generated, WB; 1:250); Rabbit polyclonal Agrin antibody, Novus Biologicals, Cat# NBP1-90209, IHC 1:50; RRID: AB_448230 (IF dilution 1:50); Anti-Integrin Beta1, activated, Clone HUTS-4, Sigma, Cat# MAB2079Z, RRID:AB_2233964 IF dilution 1:50; Mouse monoclonal anti-β-Actin (C4), Santa Cruz Biotechnology, Cat#sc-47778; RRID: AB_2714189 WB dilution 1:1000; Rabbit polyclonal anti-GAPDH (FL-335), Santa Cruz Biotechnology, Cat#sc-25778 WB Dilution 1:1000; RRID: AB_10167668; Rabbit polyclonal anti-CD31, Abcam, Cat#ab28364; RRID: AB_726362 IF-IHC 1:50; Goat anti-rabbit IgG-HRP, Santa Cruz Biotechnology, Cat#sc-2030; RRID: AB_631747 WB dilution 1:2000; Goat anti-mouse IgG-HRP, Santa Cruz Biotechnology, Cat#sc-2005; RRID: AB_631736; Goat anti-mouse IgM-HRP, Santa Cruz Biotechnology, Cat#sc-2973; RRID: AB_650513 WB dilution 1:2000; Goat anti-Rabbit IgG (H + L) Alexa Flour 488 Invitrogen, Cat#A11034 IF dilution 1:500; Goat anti-mouse IgM Alexa Flour 594, Invitrogen, Cat#A21044 (IF dilution 1:500). EGFR Monoclonal antibody, Protein tech; 66455-1-Ig; RRID: AB_2881824; EGF Receptor (C74B9), Cell Signaling Technology, #2646; RRID:AB_2230881; EGF Receptor (L858R Mutant Specific) (43B2) Cell Signaling Technology, #3197, RRID:AB_1903955; EGF Receptor (D1P9C) Rabbit mAb (Target: Mouse Preferred), Cell Signaling Technology, #71655; RRID:AB_2799807; Phospho-EGF Receptor (Tyr1068) (D7A5) Cell Signaling Technology, #48576; YAP (D8H1X) Cell Signaling Technology #14074;

RRID :AB_2650491;YAP/TAZ(D24E4), Cell Signaling Technology, #8418, RRID:AB_10950494; Phospho-YAP (Ser127) (D9W2I), Cell Signaling Technology,#13008, RRID:AB_2650553; CTGF (D8Z8U), Cell Signaling Technology #86641, RRID: AB_2800085;CYR61 (D4H5D) Cell Signaling Technology #14479, RRID: AB_11217435; Integrin β1 (D2E5); Cell Signaling Technology #9699, RRID: AB_11178800; Integrin β1 [12G10] Abcam #ab30394, RRID: AB_775726. Anti-pTyr357 YAP (4645), Sigma, RRID: AB_1080624; The

pLATS S909 antibody, Cell Signaling Technology (#9157) RRID:AB_2133515. The following siRNAs have been used: On-TARGETplus non-targeting (D-001810-10-05), human Agrin smartpool (Cat# L-031716-00-0050), human Agrin stealth siRNA (Invitrogen, cat# 1299001), human EGFR smartpool (L-003114-00-0010), human YAP1 smartpool (L-012200-00-0020), human WWTR1 (TAZ) smart pool (L-016083-00). The shRNA sequences are presented in Table S1. Lipofectamine RNAimax (Invitrogen) was used for siRNA transfections following the manufacturer's recommended guidelines. Fibronectin was obtained from Gibco, and Advanced Biomatrix. Rat tail collagen type 1 was from Corning. Osimertinib was from MedChemExpress (HY-15772); VT107 from MedChemExpress (HY-134957); AG1478 from MedChemExpress (HY-13524); Cyclo-RGD inhibiting peptide (HY-P2300), blebbistatin (HY-13813), defactinib (HY-12289) and GW5074 (HY-10542) were purchased from MedChemExpress, Human EGF ELISA kit was from Proteintech and analyzed as per manufacturer's recommended protocol.

Orthotopic cancer cell injection procedure

Mice were anaesthetized with 5% isoflurane for induction and then 2–3% for maintenance via a nose cone; mice were placed on a heating pad and positioned in right lateral decubitus for intrathoracic administration of H1299del19 cells into the left lung lobe. To assure aseptic skin puncture, the fur was clipped on the left chest and shoulder region using a cordless electric clipper. A 30-G needle attached to a 0.3 ml insulin syringe was used for administering 1×10^6 cells mixed with Matrigel (1:1) in a total volume of 50 µl to the left lung. The needle was inserted at a 90° angle at a depth of 3–5 mm caudal from the caudal angle of the scapula into the left lung. This will serve as an anatomic landmark to identify the injection site (roughly the fourth or fifth intercostal space). Visualization of the caudal angle of the scapula will be done by elevating the left forelimb. This maneuver will be conducted without changing the position of the mouse. The insertion of the needle will be performed on the cranial edge of the rib to prevent injury of subcostal nerves and vessels. After injection of the lung tumor cells, the needle was quickly removed to minimize the risk of pneumothorax. To avoid "tumor-cells spillage" to the contralateral lung, the mouse was positioned in left lateral decubitus until recovery from isoflurane anesthesia.

RNA interference and knockdown

The siRNAs were dissolved as per the manufacturer's recommended buffers. For in vitro studies, 50 nM of control or targeted siRNA was mixed with 5 µL of lipofectamine RNAimax (Thermo Fisher Scientific) and incubated for 30 min at room temperature. The siRNA:lipofectamine mixture was added to 250 µL of reduced growth factor medium. The knockdown was verified by RT-PCR or Western blot after 72 h.

Recombinant sAgrin expression and purification

The C-terminus fragment containing an eight amino acid "Z8" insert and LG3 domain was cloned into a pET28 vector containing His-tag and expressed in *Escherichia coli* (strain BL21-DE3) as previously published(*1, 2*). Western blotting and co-immunoprecipitation followed.

Western blotting and Co-immunoprecipitation

Indicated cells post-manipulation were washed twice with cold PBS and lysed with cold 1% NP-40 lysis buffer supplemented with a 1× protease inhibitor cocktail (Roche Applied Biosciences) for 15 min at 4°C. The cell lysate was centrifuged at 18,900 × g for 15 min. This was followed by protein estimation using a Pierce protein detection kit. Subsequently, 40–50 µl of total protein was mixed with an equal volume of 2X Laemmli sample buffer and heated at 95°C for 5 min. This was followed by resolution with SDS–PAGE gel. The resolved proteins were transferred onto nitrocellulose membranes and blocked in 5% skimmed milk reconstituted in 1× PBS containing 0.1% Tween-20 and probed overnight with the respective primary antibody. The membrane was then washed with 1× PBS supplemented with 0.1% Tween-20: three times at 15 min intervals. This was followed by 1 h incubation in conjugated horseradish peroxidase (HRP) secondary antibody (Santa Cruz Biotechnology). Post-incubation, the blot was again washed three times as above and then overlaid with enhanced chemiluminescence (ECL) substrate (Pierce/Bio-Rad) and visualized on X-ray film by using an image processor or digitally by a Chemidoc analyzer (Bio-Rad), with data analysis via Image Lab 6.0.1 software. The density of the various bands was quantified using Image-J 1.52e software. For tumor tissues, snap-frozen tissues were first crushed in a pre-chilled mortar pestle and homogenized for 10 cycles. Further, 300–600 µL of ice-cold lysis buffer (RIPA, with

added protease inhibitor cocktail) was added to 1.5 ml tumor lysate and samples were subjected to shaking for 2 h at 4°C. The tubes were centrifuged at 16,000 × g for 20 min at 4°C, and then, the supernatant was collected, protein content estimated, and samples were further processed for Western blots as above.

For co-immunoprecipitation assays, cells were lysed in NP-40 lysis buffer (20 mM Tris-HCl, pH 7.5, 150 mM NaCl, 1% Triton X-100, and 1 mM PMSF [phenylmethylsulfonyl fluoride] with complete EDTA-free protease inhibitor mixture [Roche]). The lysate was incubated on ice for 30 min and cleared by centrifugation at 13,000 rpm for 30 min at 4°C. Immunoprecipitation was performed at 4°C with 4 µg of antibody in the presence of either protein A or protein G–Sepharose 4 Fast Flow (GE Healthcare) for 4 h at 4°C in a rocker. The supernatants containing the sepharose bound proteins were then washed three times with lysis buffer and three times with cold PBS. Bound proteins were eluted with 2× Laemmli sample buffer and resolved by SDS-PAGE (sodium dodecyl sulfate–polyacrylamide gel electrophoresis) for subsequent Western blotting.

Immunofluorescence and confocal microscopy

Cells were cultured on eight-well chamber slides or coverslips overnight. Cells were then washed twice with PBS and fixed for 15 min with 4% paraformaldehyde. Subsequently, the cells were permeabilized for 15 min with 0.1% Triton X in PBS containing 1 mM Ca^{+2} and 1 mM Mg^{+2} (PBSCM) at room temperature. The permeabilized cells were incubated with indicated antibodies in fluorescent dilution buffer (FDB) for 1–2 h at room temperature or overnight at 4°C, followed by five washes with PBSCM and incubation with secondary antibody; Alexa Fluor (Thermo Fisher Scientific) for 1 h at room temperature. Slides were again washed five times with PBSCM and mounted with Vectashield medium containing DAPI. The stained cells were then imaged by a Zeiss or Olympus confocal microscope. The images were processed and analyzed by Zen blue (Zen 2 lite) or CellSense Standard software.

Molecular Docking Simulation

The respective PDB-generated crystal structures of the extracellular domains (ECD) of EGFR (amino acid residues 25-645), integrin β1 (residues: 21-738) were juxtaposed with sAgrin (residues 1617-2068) using

Alphafold 3.0 prediction modeling on ChimeraX (UCSF) platform. The respective cartoon for Alphafold polymeric and the surface plot of the trimeric protein interaction are presented.

Recombinant Protein Fc-Pulldown assay

Human EGFR (amino acids 1-645) tagged with C-terminal human IgG1 Fc and Integrin β1 corresponding to amino acids 21-738 containing a C-terminal poly-Histidine tag were expressed in HEK293 (Sino Biological). Purity of isolated recombinant proteins were >95% as assessed by SDS-PAGE and HPLC. Two hundred µg of EGFR was immobilized to 40 µl Protein-A Agarose Beads (Sigma-Aldrich) for 2 h. Subsequently after washes, the beads were incubated with Integrin β1 in the presence or absence of sAgrin. The resultant complex was resolved in an SDS-PAGE gel.

Immunohistochemistry

The mouse lungs were fixed in 10% Neutral-buffered formalin (NBF) for 24 h. The lung tissues were processed, embedded and sectioned using a microtome at 5 micrometers. The slides were deparaffinized and rehydrates in graded alcohol and subjected to antigen-retrieval in citrate buffer at pH 9 or pH 6 (for the indicated antibodies). The slides were blocked in 3% hydrogen peroxide followed by 10% goat serum or rabbit serum (depending on the antibodies), followed by Avidin/Biotin block. They were stained with primary antibodies for 30 min followed by 3-5 1X PBS washes for 3-5 min each. They were stained with secondary antibodies for 15 min, and subsequently covered by a coverslip. The slides were visualized under a brightfield microscope. The following primary antibody dilutions were used for IHC: Agrin-Invitrogen #PA5-37121 1:400; Agrin-Abcam #ab85174 1:1000; YAP-Cell Signaling #14074S 1:400; TAZ- Cell Signaling #72804S 1:400; CD34-Novus Bio #600-1071 1:1000; KI67-Abcam #ab15580 1:2500; Caspase 3-Cell Signaling Cell Signaling #9664 1:400; CD31-Abcam #ab28364 1:50. Secondary antibodies (Vector labs): Anti Mouse BA2000 1:1000; Anti Rabbit BA1000 1:600; Anti Rat BA9400 1:600; Immunofluorescence stain: Anti-Integrin beta 1/pEGFR, 1:500/1:700 Abcam #ab30394/Cell Signaling #48576SF Alexa Fluor 555/Alexa Fluor 488 1:1000/1:1000.

Cytotoxicity assay

The survival rate of cells treated with VT-107, OSI, and a combination of both, compared with the control group (DMSO, 0.002 μM), was assessed. This was achieved by incorporating the AlamarBlue reagent (sourced from AbD Serotec, MorphoSys, Raleigh, NC, USA), constituting 10% of the total volume, into each well. The mixture was incubated for 4 hours prior to the measurement of fluorescence using the SPECTRAmax Gemini Spectrophotometer, with an excitation wavelength of 540 nm and emission wavelength of 590 nm. The relative IC50 (half-maximal inhibitory concentration) values were calculated by plotting the surviving percentage of cells versus the concentration of individual inhibitors using Graphpad Prism 9.0.

Quantitative reverse transcription PCR (RT-PCR)

Total RNA was extracted from indicated cells using Qiagen RNeasy mini kit as per the manufacturer's recommended protocol described as follows. The total RNA was then reverse-transcribed using a High-Capacity cDNA reverse transcription kit (Applied Biosystems). The generated cDNA (200 ng) was used as a template for the RT-PCR using SYBRTMGreen or TaqmanR (Thermo Fisher Scientific) based master mix and probes. The data were normalized to GAPDH as an endogenous control. The RT-PCR primers were used in the study for the respective target genes as noted in Supplementary Table 2.

Colony formation assay and 3D cultures in soft and stiff ECM

Twenty thousand cancer cells with indicated manipulations were plated on 0.5 kPa or 30 kPa coated stiffness plates, 12 wells plates (Cytosoft, Inc), in 600 μL of complete growth medium. For sAgrin treatment, wells were treated with 10 μg/ml or indicated concentrations every two days in complete medium. The colonies that developed at the end of seven days were fixed in ice-cold methanol, washed in PBS (5×), and subsequently stained by 0.5% Crystal Violet solution for 30 min at room temperature. The representative wells were imaged under a bright-field microscope. For 3D-stiffness-cultures, 10,000 PC9/H1975 cells were resuspended in 300 μL of VitroGel® solution amounting to highly stiff (>30 kPa) conditions or in a diluted VitroGel®:media solution containing 60 μL of VitroGel® and 240 μL of complete medium that generated a soft matrix. The mixture was

immediately added to eight well chamber slides (Nalge-Nunc) and cultured for 3–4 days. sAgrin was added in the respective wells both during gelation as well as in the medium after the gelation process was achieved.

Bioinformatic analysis of clinical data

To ascertain agrin as a co-expressed gene with EGFR mutation, we analyzed the Cancer Genome Atlas (TCGA) cohort of LUAD (Lung adenocarcinoma_Nature 2014 and Lung adenocarcinoma Firehose Legacy) to assess the EGFR mutational status, agrin expression, and the impact of overall survival in these cohorts. These genes were further stratified for ECM-associated signatures using Matrisome AnalyzerR (UIC). For analysis of agrin with YAP/TAZ in LUAD, we further analyzed the TCGA_LUAD dataset with 515 patients with both clinical and gene expression data. Among them, only 37 were known to have received targeted therapy. In this report, we analyzed the association of AGRN and YAP/TAZ genes with overall survival (OS) in the entire cohort (n = 515). Using a multivariable Cox regression with all YAP/TAZ genes and stepwise feature selection by AIC (Akaike information criterion), we identified genes that were significantly associated with OS.

Clinical specimens

We have used a broad range of lung tumors from patients (n>125 patients) of different geographical and demographic regions. First, we stained for EGFR and Agrin co-expression in a commercially available LUAD tissue microarray (TMA) (Tissue Array, LC10014a) consisting of 50 cases of LUAD from different stages. Second, we also stained a lung cancer TMA STR 192-14 from Singapore General Hospital (SGH) comprising of 49 cases of lung cancers (30-adenocarcinoma, 14-squamous cell carcinoma, 5-other types of lung cancers). These patients were mainly of Chinese origin. Third cohort consisted of 27 LUAD patients who were largely Caucasians and treated at Roswell Park Comprehensive Cancer Center. Among these samples, 17 contained EGFR mutations, 5 were negative of EGFR mutations and 5 were not tested.

Supplemental references:

1. S. Chakraborty *et al.*, Agrin-Matrix Metalloproteinase-12 axis confers a mechanically competent microenvironment in skin wound healing. *Nat Commun* **12**, 6349 (2021).
2. K. Njah *et al.*, A Role of Agrin in Maintaining the Stability of Vascular Endothelial Growth Factor Receptor-2 during Tumor Angiogenesis. *Cell Rep* **28**, 949-965 e947 (2019).
